# Distinct contributions of O‐acetylserine sulfhydrylases to cysteine biosynthesis in *Pseudomonas aeruginosa*

**DOI:** 10.1002/pro.70498

**Published:** 2026-02-12

**Authors:** Noemi Massa, Flavia Catalano, Silvia Fruncillo, Francesca Troilo, Marta Mellini, Filippo Favretto, Livia Leoni, Giordano Rampioni, Alessandro Giuffrè, Adele di Matteo, Alessandra Astegno

**Affiliations:** ^1^ Department of Biotechnology University of Verona Verona Italy; ^2^ CNR Institute of Molecular Biology and Pathology Rome Italy; ^3^ Department of Science University Roma Tre Rome Italy; ^4^ IRCCS Fondazione Santa Lucia Rome Italy

**Keywords:** cysteine biosynthesis, O‐acetylserine sulfhydrylase, protein‐peptide interaction, *Pseudomonas aeruginosa*, steady‐state and pre‐steady‐state kinetics, substrate specificity

## Abstract

Cysteine biosynthesis in bacteria proceeds primarily via the *de novo* pathway, involving serine acetyltransferase (CysE) and O‐acetylserine sulfhydrylase (OASS). This pathway is absent in humans, and its inhibition impairs microbial fitness, virulence, and antibiotic resistance, making its enzymes attractive antimicrobial targets. Most bacteria encode two OASS isoforms: CysK, which forms the cysteine synthase complex (CSC) with CysE, and CysM, which typically acts independently. While conserved, their biochemical properties and regulatory roles vary across species. Here, we investigated CysK (PaCysK) and CysM (PaCysM) from *Pseudomonas aeruginosa*, an opportunistic pathogen of major concern due to intrinsic antibiotic resistance. We characterized their steady‐state and pre‐steady‐state kinetics, structural features, and assessed substrate preferences through microbiological analyses of *cysK* and *cysM* deletion mutants. Our results revealed that PaCysK and PaCysM play redundant yet critical roles in cysteine biosynthesis in *P. aeruginosa*. PaCysK exhibits optimal activity with sulfide, supporting its primary function in sulfide‐dependent cysteine biosynthesis. In contrast, PaCysM shows broader specificity, catalyzing cysteine formation from both sulfide and thiosulfate, suggesting a specialization for alternative sulfur sources. Structural modeling supports this, revealing PaCysM active‐site features that facilitate thiosulfate binding and turnover. We also tested PaCysK binding to a synthetic peptide mimicking the C‐terminal region of *P. aeruginosa* CysE1 and found no interaction, suggesting that the CSC does not form in *P. aeruginosa*. In contrast, PaCysK binds the equivalent peptide from *Salmonella enterica* serovar Typhimurium CysE, known to mediate CSC formation, confirming that the enzyme retains the capacity for protein–protein interactions through a canonical CSC‐like mechanism.

## 1 INTRODUCTION

Cysteine is a sulfur‐containing amino acid essential for numerous cellular processes. It plays a critical role in protein structure, glutathione biosynthesis, redox homeostasis, and enzymatic activity, with its thiol group contributing to the formation of iron–sulfur clusters involved in metabolism and DNA repair.

**FIGURE 1:**
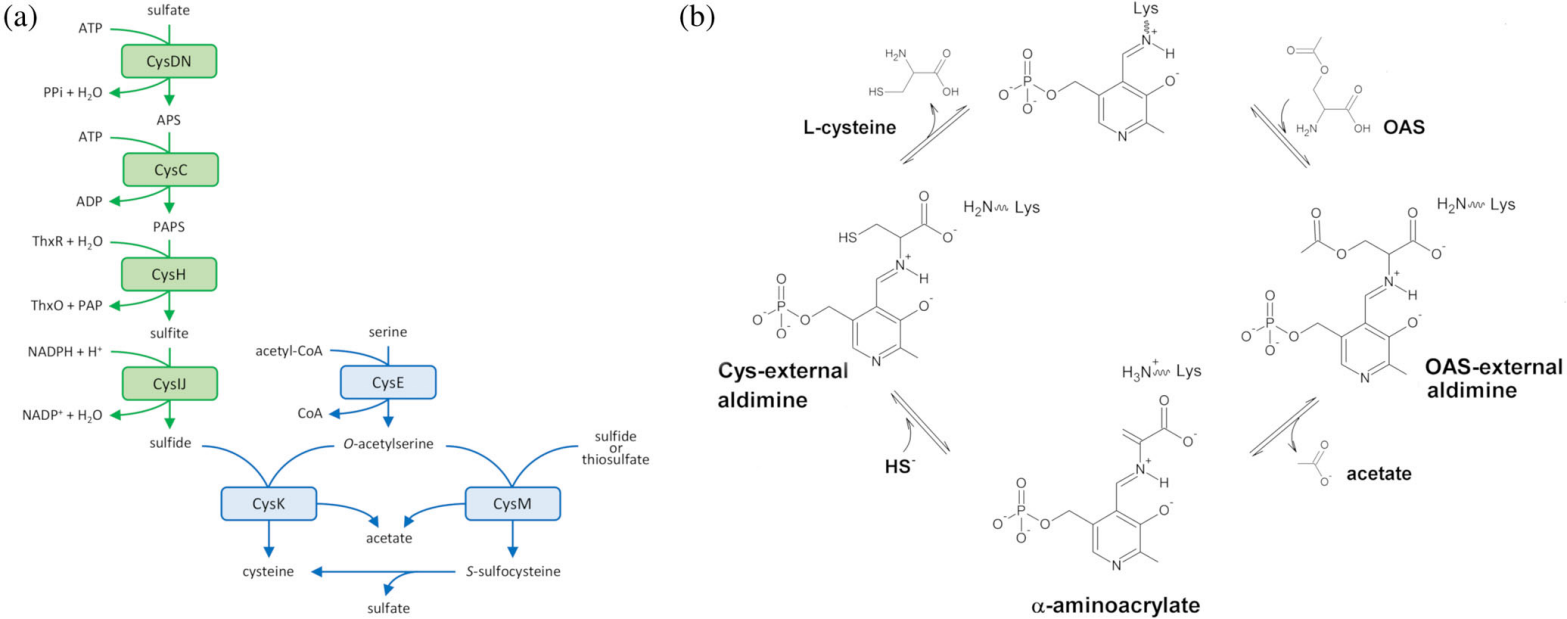
Pathways of sulfate reduction and cysteine biosynthesis in *E. coli*, and the reaction mechanism of OASS. (a) Sulfate reduction (in green) and *de novo* cysteine biosynthetic (in blue) pathway in *E. coli*. ATP, adenosine triphosphate; PPi, inorganic phosphate; APS, adenosine 5′‐phosphosulfate; ADP, adenosine diphosphate; PAPS, 3′‐phosphoadenosine 5′‐phosphosulfate; ThxR, reduced thioredoxin; ThxO, oxidized thioredoxin; PAP, adenosine 3′,5′‐bisphosphate; NADPH, reduced nicotinamide adenine dinucleotide phosphate; NADP^+^, oxidized nicotinamide adenine dinucleotide phosphate; CoA, coenzyme A. CysDN, sulfate adenylyltransferase (EC 2.7.7.4); CysC, APS kinase (EC 2.7.1.25); CysH, PAPS reductase (EC 1.8.4.8); CysIJ, NADPH sulfite reductase (EC 1.8.1.2); CysE, serine acetyltransferase (SAT; EC 2.3.1.30); CysK, *O*‐acetylserine sulfhydrylase (OASS‐A; EC 2.5.1.47); CysM, *O*‐acetylserine sulfhydrylase (OASS‐B; EC 2.5.1.47). Image modified from (Sekowska et al., 2000). (b) Catalytic mechanism for OASS. Image modified from (Mozzarelli et al., 2011).

In bacteria, the cysteine biosynthesis involves intricate and often interconnected pathways. In *Escherichia coli*, where this process has been well‐characterized, the primary route for *de novo* cysteine biosynthesis is the sulfate assimilation pathway (Figure 1a; Guédon & Martin‐Verstraete, 2007). This process begins with the uptake and activation of inorganic sulfate, followed by its sequential reduction to bisulfide. The final two steps of this *de novo* pathway are catalyzed by two key enzymes: serine acetyltransferase (SAT/CysE; EC 2.3.1.30) and O‐acetylserine sulfhydrylase (OASS; EC 2.5.1.47). Notably, this pathway is absent in mammals, which obtain cysteine from the diet or via the transsulfuration pathway converting methionine to cysteine (Sbodio et al., 2019). This aspect, together with the observation that the enzymes of the *de novo* cysteine biosynthetic pathway are required for growth, also in cysteine‐containing media, and for full virulence in several bacterial pathogens (Hicks et al., 2022), highlights cysteine biosynthetic enzymes as attractive targets for antibacterial strategies (Brunner et al., 2016; Burns‐Huang & Mundhra, 2019; Turnbull & Surette, 2008; Turnbull & Surette, 2010).

Among these two enzymes, CysE catalyzes the first and often rate‐limiting step in the *de novo* pathway. It transfers an acetyl group from acetyl‐coenzyme A to L‐serine, yielding O‐acetylserine (OAS) and CoA (Figure 1a). This step is regulated at multiple levels. CysE is allosterically inhibited by cysteine, providing a feedback mechanism to prevent overaccumulation of the amino acid. Moreover, the transcription of *cysE* and other genes involved in sulfur metabolism is regulated by intracellular sulfur levels, often mediated by the transcriptional regulator CysB (Campanini et al., 2015; Francois et al., 2006; Salsi, Campanini, et al., 2010). The second step in cysteine biosynthesis is catalyzed by OASS, also known as cysteine synthase (Figure 1a). OASS is a pyridoxal 5'‐phosphate (PLP)‐dependent enzyme that typically assembles into dimers or tetramers and belongs to the family of type II PLP‐dependent enzymes. Its overall fold is characterized by two α/β domains, with the active site located in the cleft formed at the interface between the N‐terminal and C‐terminal domains, where the PLP cofactor is anchored through a conserved catalytic lysine residue (Bettati et al., 2000; Burkhard et al., 1998; Raj et al., 2012). Functionally, OASS follows a ping‐pong kinetic mechanism to catalyze sulfur incorporation into OAS (Campanini et al., 2015). This reaction proceeds through a β‐elimination of acetate from OAS, yielding an α‐aminoacrylate intermediate that subsequently undergoes nucleophilic attack by a sulfur donor to form L‐cysteine (Figure 1b; Mozzarelli et al., 2011). In the resting state, the enzyme is in the internal aldimine form, in which the PLP is bound to the catalytic lysine residue. Upon OAS binding, an external Schiff base is formed, enabling the β‐elimination step that generates the α‐aminoacrylate intermediate. Sulfide then attacks the β‐carbon, reprotonation of the α‐carbon occurs, and cysteine is released following regeneration of the internal aldimine. In‐depth mechanistic studies of OASS have also identified additional transient species, including geminal‐diamine intermediates and tautomeric forms of both internal and external Schiff bases (Chattopadhyay et al., 2007; Rabeh & Cook, 2004; Schnell et al., 2007).

In many bacteria, two distinct OASS isoforms exist: OASS‐A (encoded by *cysK*) and OASS‐B (encoded by *cysM*). OASS‐A is the most extensively characterized isoform (Becker et al., 1969; Burkhard et al., 1998; Cook & Wedding, 1976; Cook & Wedding, 1977; Kredich et al., 1969; Raj et al., 2012; Tai et al., 1993) and is typically the major, constitutively expressed enzyme for cysteine production. In contrast, the physiological role of CysM remains controversial and is yet to be fully elucidated (Mozzarelli et al., 2011). Importantly, CysK, but not CysM, can interact with CysE to form the cysteine synthase complex (CSC), a regulatory assembly that is stabilized by the insertion of the C‐terminal region of CysE into the active site of CysK, where it competitively inhibits the enzyme activity (Huang et al., 2005; Kredich et al., 1969; Rabeh & Cook, 2004; Salsi et al., 2010; Schnell et al., 2007; Zhao, Moriga, et al., 2006). In species where the CSC forms, the C‐terminal region of CysE typically ends with a conserved isoleucine residue, which plays a central role in the interaction. This isoleucine contributes approximately 80% of the total binding energy and acts as the main anchoring element that ensures the correct positioning of CysE within the CysK active site (Cook & Wedding, 1976; Huang et al., 2005; Zhao, Moriga, et al., 2006). CSC formation and dissociation are dynamically regulated by pathway intermediates; high levels of OAS promote complex dissociation by competing with CysE for binding at the CysK active site (Kredich et al., 1969; Schnell et al., 2007; Wang & Leyh, 2012; Wirtz et al., 2001), whereas sulfide stabilizes the interaction, likely serving as a signal of adequate sulfur availability.

Intriguingly, beyond its role in cysteine biosynthesis, CysK has been implicated in various moonlighting functions in bacterial physiology. These include the activation of contact‐dependent toxins in some Gram‐negative pathogens (Diner et al., 2012; Johnson et al., 2016), the modulation of the cysteine metabolism regulator CymR by stabilizing CymR‐DNA interaction in *Bacillus subtilis* (Tanous et al., 2008), and the alteration of biofilm formation in *Aliivibrio fischeri* (Singh et al., 2015). Several of these activities depend on CysK binding to protein partners, with complex formation modulated by OAS and/or sulfide availability.

*Pseudomonas aeruginosa*, an opportunistic human pathogen known for its metabolic versatility and intrinsic antibiotic resistance, encodes homologs of *E. coli* CysK and CysM along with two isoforms of CysE (CysE1 and CysE2) potentially involved in cysteine production. Furthermore, unlike many bacteria, *P. aeruginosa* has the enzymes required to convert homocysteine to cysteine via the transsulfuration pathway, involving cystathionine β‐synthase (CBS) and γ‐cystathionase (CGL; Caruso et al., 2024; Pedretti et al., 2024). This metabolic duality, encompassing both the* de novo* synthesis and the reverse transsulfuration pathway, raises fundamental questions about how cysteine metabolism in *P. aeruginosa* might differ from that in other bacteria and, critically, from its mammalian host.

In this study, we employed an integrated approach combining enzyme kinetics, structural modeling, microbiological assays, and protein–protein interaction analyses to characterize the two OASS isoenzymes CysK (PaCysK) and CysM (PaCysM) from *P. aeruginosa* and to define their respective roles in cysteine biosynthesis. Kinetic analyses under steady‐state and pre‐steady‐state conditions indicate that PaCysK is primarily adapted for sulfide‐dependent cysteine biosynthesis, whereas PaCysM exhibits broader sulfur donor utilization with a pronounced preference for thiosulfate. This functional differentiation is reflected in LC–MS/MS product profiles and is further supported by structural features of PaCysM that are compatible with the accommodation of alternative sulfur donors. Microbiological analysis of *cysK* and *cysM* deletion mutants showed that PaCysK and PaCysM play key but partially redundant roles in cysteine biosynthesis, as simultaneous loss of both genes nearly abolished growth in cysteine‐free minimal medium. Growth phenotypes further indicated that PaCysK primarily supports sulfide‐dependent cysteine biosynthesis, whereas PaCysM is specialized for thiosulfate utilization, consistent with their distinct catalytic properties.

The regulatory potential of PaCysK was also examined through protein–protein interaction assays. We found that PaCysK does not interact detectably with a peptide corresponding to the C‐terminal region of PaCysE1, which lacks the conserved isoleucine required for CSC formation, indicating that canonical CSC‐mediated regulation is unlikely to operate in *P. aeruginosa*. Nevertheless, PaCysK retained the ability to recognize a canonical CysE‐like motif, as demonstrated by its interaction with a model peptide derived from the C‐terminal region of *Salmonella enterica* serovar Typhimurium CysE, which is known to form the CSC. No comparable interaction was observed for PaCysM. These results suggest that PaCysK preserves the intrinsic capacity for CSC‐like interactions, even though the endogenous *P. aeruginosa* CysE isoforms do not appear to exploit this regulatory mechanism.

Overall, this work demonstrates how the coexistence of two OASS isoenzymes in *P. aeruginosa* reflects a finely balanced interplay between functional redundancy and specialization, with important implications for the regulation and potential therapeutic targeting of cysteine biosynthesis.

## 2 RESULTS

### 2.1 Production and biochemical characterization of PaCysK and PaCysM

*P. aeruginosa* PAO1 harbors two isoforms of OASS, encoded by the *cysK* and *cysM* genes. Sequence analysis of the two isoenzymes (PaCysK, UniProt ID: Q9I0D3; PaCysM, UniProt ID: Q9I526) revealed approximately 41% amino acid identity, indicative of a shared catalytic ancestry. Comparative sequence analysis with OASS orthologs from other microorganisms, including *S. enterica* serovar Typhimurium (St), *Escherichia coli* (Ec), *Haemophilus influenzae* (Hi), and *Mycobacterium tuberculosis* (Mt), highlighted the broad conservation of OASS catalytic domains across bacterial species, while also reflecting lineage‐specific adaptations (Figure S1). Notably, PaCysK shares 70.8% amino acid identity with StCysK, 66.6% with HiCysK, 71.3% with EcCysK, and 55.7% with MtCysK1. Similarly, PaCysM exhibits 68.8% amino acid identity with both EcCysM and StCysM, and 39% with MtCysM.

**FIGURE 2:**
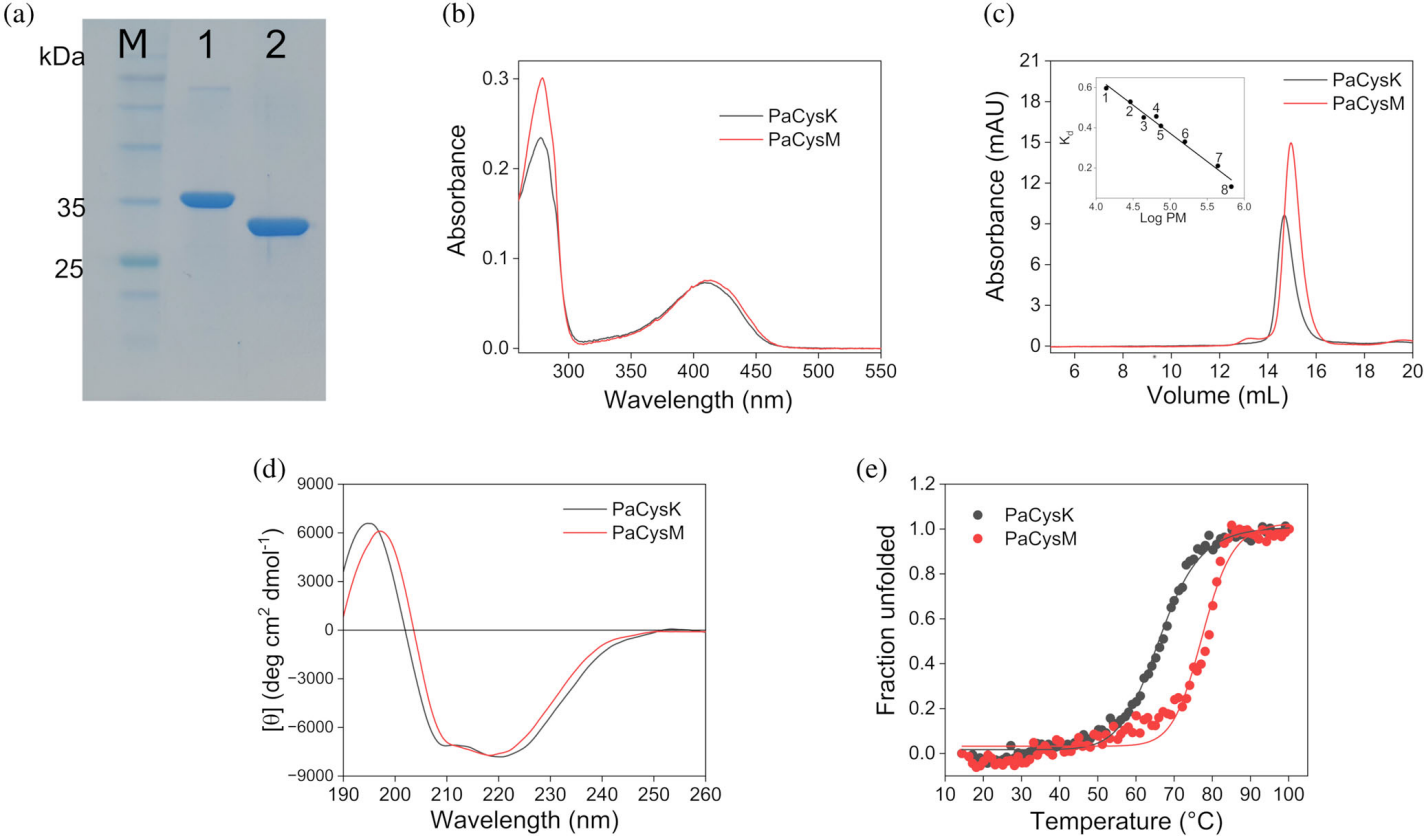
Biochemical properties of PaCysK and PaCysM. (a) 12% SDS‐PAGE of purified recombinant proteins. Lane M, protein marker; lane 1, PaCysK; lane 2, PaCysM. (b) UV–visible absorption spectra of 15 μM PaCysK (black) and PaCysM (red) recorded in 20 mM sodium phosphate (NaP), 150 mM NaCl, 0.1 mM DTT, pH 8.5. (c) Size exclusion chromatography of PaCysK (black) and PaCysM (red) at 1 mg mL^−1^ using a Superdex 200 GL 10/300 column in 50 mM Hepes, 150 mM NaCl, 0.1 mM DTT, pH 8.5. Inset, calibration curve of logarithm of the MW versus distribution coefficient. The standard proteins used were: 1, ribonuclease A; 2, carbonic anhydrase; 3, ovalbumin; 4, aprotinin; 5, conalbumin; 6, aldolase; 7, ferritin; 8, thyroglobulin. (d) Far‐UV CD spectra of 0.2 mg mL^−1^ PaCysK (black) and PaCysM (red) in 20 mM NaP pH 8. (e) Thermal denaturation of PaCysK (black) and PaCysM (red) recorded following ellipticity signal at 222 nm in the same conditions as in (d). Fitting the curve with a sigmoidal function resulted in Tm_app_ ~66°C for PaCysK and ~77°C for PaCysM.

PaCysK and PaCysM were successfully overexpressed in *E. coli* and purified as His‐tagged proteins to >95% purity, as assessed by SDS‐PAGE (Figure 2a). The recombinant proteins displayed a characteristic yellow coloration and exhibited UV–visible absorption spectra with a prominent peak at ~412 nm, consistent with the presence of the ketoenamine tautomer of the PLP internal aldimine (protein‐PLP complex; Figure 2b). The difference in the absorbance ratios at 280/412 nm for PaCysK (3.2) and 280/414 nm for PaCysM (4.0) is consistent with the higher number of tyrosine residues in PaCysM (9) compared to PaCysK (5), despite both isoenzymes containing two tryptophan residues. Both PaCysK and PaCysM bind one mol of PLP per mole of monomer.

Size‐exclusion chromatography indicated that both proteins predominantly exist as dimers in solution, with estimated molecular weights of ~71 kDa for PaCysK and ~67 kDa for PaCysM, consistent with their respective monomeric molecular masses of ~35.3 and 33.5 kDa (Figure 2c). Far‐UV (190–260 nm) circular dichroism (CD) analysis revealed nearly superimposable spectra with the characteristic minima near 210 and 222 nm, indicating predominantly α‐helical secondary structure content (Figure 2d). Moreover, thermal denaturation experiments monitored by CD ellipticity at 222 nm revealed cooperative unfolding, with apparent melting temperatures (Tm_app_) of ~66°C for PaCysK and of ~77°C for PaCysM (Figure 2e). Overall, these findings demonstrate that both recombinant enzymes are properly folded and structurally stable, with PaCysM exhibiting significantly higher thermal stability than PaCysK.

### 2.2 Steady‐state kinetic properties of PaCysK and PaCysM

**FIGURE 3:**
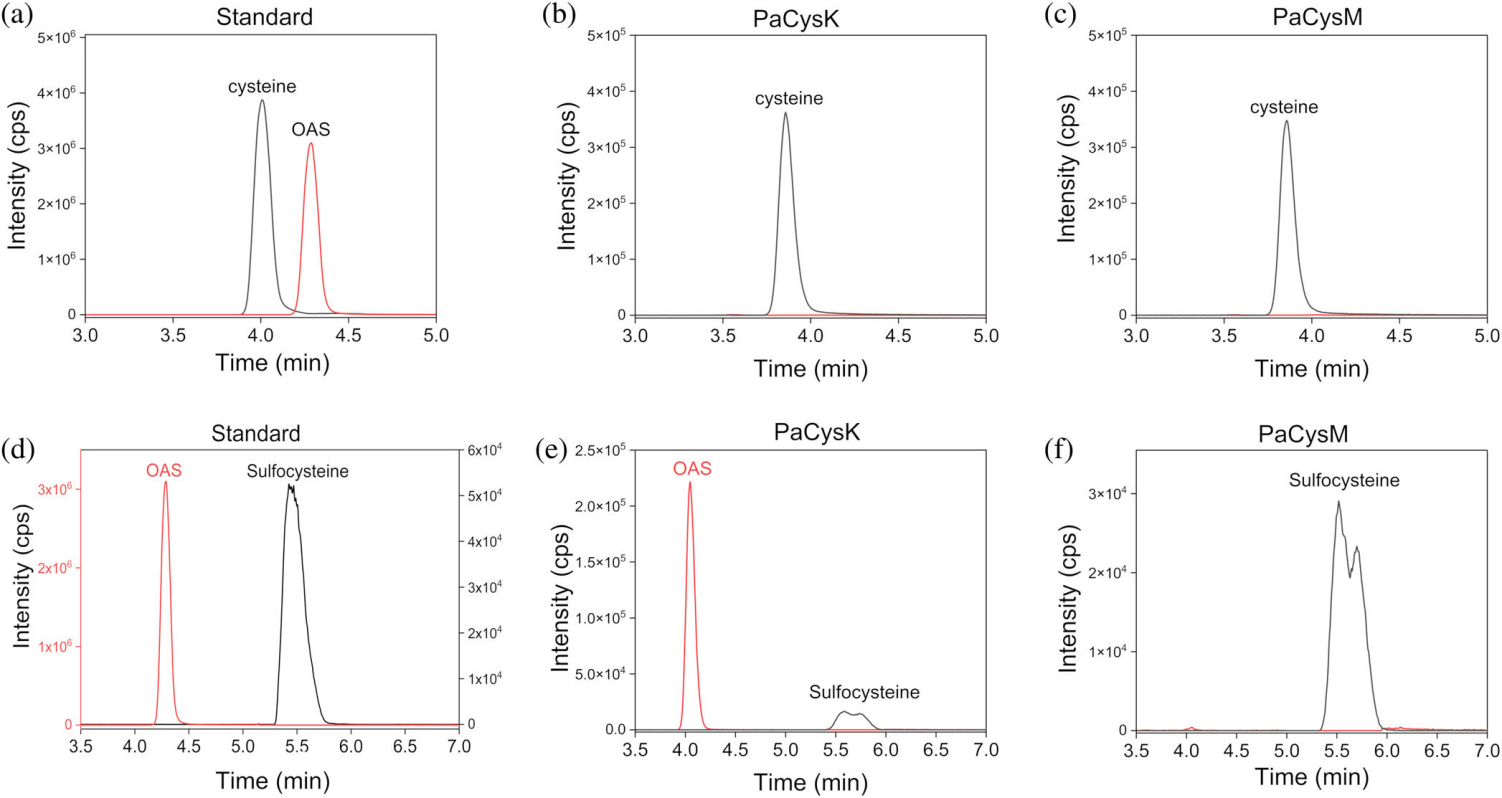
LC–MS/MS product analysis of PaCysK‐ and PaCysM‐catalyzed reactions using Na_2_S or Na_2_S_2_O_3_ as sulfur donor. LC–MS/MS chromatograms of reaction products obtained in the presence of 10 mM OAS and 5 mM Na_2_S (a–c) or 5 mM Na_2_S_2_O_3_ (d–f). Reactions were carried out at 37°C for 30 min using 15 μM PaCysK or PaCysM in 10 mM Hepes, pH 8.0.

To determine whether PaCysK and PaCysM are functionally active, we performed a qualitative analysis of reaction products by liquid chromatography–tandem mass spectrometry (LC–MS/MS). Specifically, *in vitro* enzymatic reactions were carried out using OAS and different sulfur donors. When OAS and Na_2_S were used as substrates (reaction: OAS + HS^−^ → L‐cysteine + acetate), both isoenzymes produced cysteine as the sole product, with no detectable residual OAS (Figure 3a–c), indicating efficient sulfide utilization. However, a clear difference emerged with sodium thiosulfate (Na_2_S_2_O_3_) as the sulfur donor (reaction: L‐OAS + S_2_O_3_^2−^ → S‐sulfocysteine + acetate; Figure 3d–f). PaCysM produced S‐sulfocysteine with complete consumption of OAS, demonstrating efficient thiosulfate utilization. In contrast, although PaCysK was also able to catalyze S‐sulfocysteine formation, reactions performed under the same conditions showed substantial amounts of unreacted OAS, indicating a markedly lower efficiency in thiosulfate‐dependent cysteine biosynthesis.

To quantify the catalytic activity of PaCysK and PaCysM, we initially employed 5‐thio‐2‐nitrobenzoate (TNB) as a chromogenic model substrate in the presence of OAS. Although TNB is not a physiological substrate, it can serve as an alternative nucleophile to sulfide by reacting with the enzyme‐bound α‐aminoacrylate intermediate to form S‐(3‐carboxy‐4‐nitrophenyl)‐L‐cysteine (Tai et al., 1993). The resulting data are shown in Figure S2, and the corresponding kinetic parameters are reported in Table S1. Both PaCysK and PaCysM were able to utilize TNB, albeit with low catalytic efficiency, indicating that both enzymes are catalytically competent. Notably, PaCysM displayed a higher catalytic efficiency than PaCysK under the conditions tested. The kinetic parameters obtained are in line with those reported for OASS homologs from other organisms, including *S. enterica* serovar Typhimurium (Table S1; Tai et al., 1993).

**FIGURE 4:**
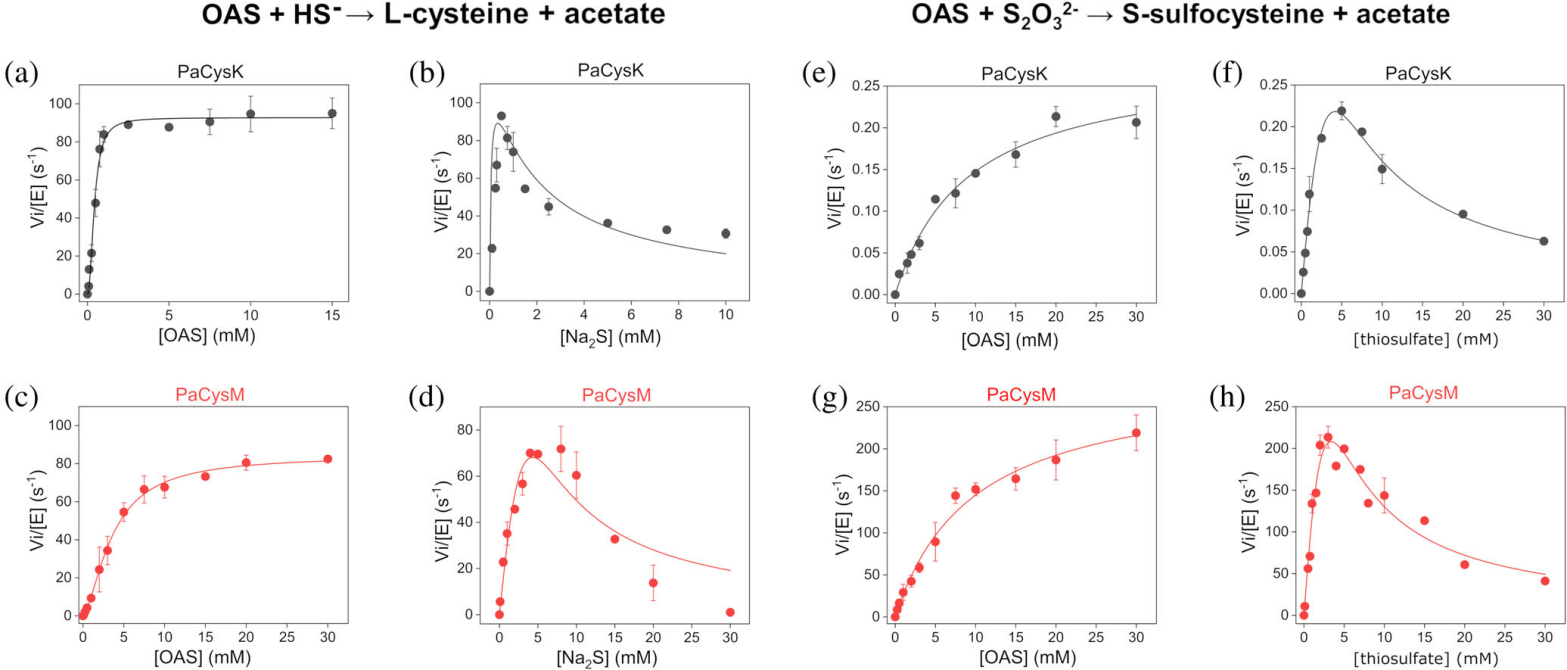
Steady‐state enzyme kinetics of PaCysK and PaCysM using Na_2_S (left panels) or Na_2_S_2_O_3_ (right panels) as sulfur donors in the presence of OAS. (a, b) PaCysK reaction rates as a function of OAS concentration (a; Na_2_S fixed at 0.5 mM) and Na_2_S concentration (b; OAS fixed at 10 mM). (c, d) PaCysM reaction rates as a function of OAS concentration (c; Na_2_S fixed at 5 mM ) and Na_2_S concentration (d; OAS fixed at 10 mM ). (e, f) PaCysK reaction rates as a function of OAS concentration (e; Na_2_S_2_O_3_ fixed at 5 mM ) and Na_2_S_2_O_3_ concentration (f; OAS fixed at 20 mM ). (g, h) PaCysM reaction rates as a function of OAS concentration (g; Na_2_S_2_O_3_ fixed at 3 mM) and Na_2_S_2_O_3_ concentration (h; OAS fixed at 20 mM). Kinetic parameters derived from these experiments are summarized in Table 1.

We subsequently performed steady‐state kinetic analyses with the physiological substrates sulfide and thiosulfate, using the ninhydrin assay. We first examined the canonical reaction using OAS and Na_2_S as substrates (Figure 4a–d and Table 1). Both enzymes exhibited similar turnover rates (*k*_cat_ = 101 ± 1 s^−1^ for PaCysK and 92 ± 1 s^−1^ for PaCysM) for the reaction. For OAS, positive cooperativity (Hill coefficient *n* ≈2) was observed in both enzymes, consistent with previously reported allosteric regulation in dimeric OASS enzymes (Bertagnolli & Wedding, 1977). Notably, PaCysK showed a higher affinity for OAS, with a *K*_0.5_ of 0.4 ± 0.1 mM compared to 3.1 ± 0.5 mM for PaCysM, resulting in a ~ 8‐fold higher catalytic efficiency for PaCysK (*k*_cat_/*K*_0.5_ ~ 2.5 × 10^5^ M^−1^ s^−1^ vs. 0.3 × 10^5^ M^−1^ s^−1^). A similar trend was observed for Na_2_S. Although both enzymes efficiently utilized sulfide, PaCysK again demonstrated substantially higher catalytic efficiency (~66‐fold greater than PaCysM), mainly due to its higher affinity for Na_2_S (*K*_*m*_ = 0.010 ± 0.003 mM for PaCysK vs. 0.6 ± 0.1 mM for PaCysM). Interestingly, both enzymes showed substrate inhibition by Na_2_S (Figure 4b,d) with inhibition constants (*K*_*i*_) of 2.1 ± 0.7 mM for PaCysK and 14 ± 3 mM for PaCysM, in agreement with previous reports (Bertagnolli & Wedding, 1977; Cook & Wedding, 1976; Kuske et al., 1994).

**TABLE 1:** Steady‐state kinetic parameters of PaCysK and PaCysM at 37°C.

	PaCysK	PaCysM
L‐OAS + HS^−^ → L‐Cys + acetate		
*k*_cat_ (s^−1^)	101 ± 1	92 ± 1
*K*_0.5_ (OAS) (mM)	0.4 ± 0.1	3.1 ± 0.5
*n*	2.4 ± 0.1	1.8 ± 0.4
*K*_*m*_ (Na_2_S) (mM)	0.010 ± 0.003	0.6 ± 0.1
*K*_*i*_ (Na_2_S) (mM)	2.1 ± 0.7	14 ± 3
*k*_cat_/*K*_*0.5*_ (OAS) (M^−1^ s^−1^)	(2.5 ± 0.6) × 10^5^	(0.30 ± 0.05) × 10^5^
*k*_cat_/*K*_*m*_ (Na_2_S) (M^−1^ s^−1^)	(1.0 ± 0.3) × 10^7^	(1.53 ± 0.26) × 10^5^
L‐OAS + S_2_O_3_^2−^ → S‐sulfocysteine + acetate		
*k*_cat_ (s^−1^)	0.29 ± 0.04	286 ± 21
*K*_*m*_ (OAS) (mM)	10 ± 4	6.1 ± 0.6
*K*_*m*_ (S_2_O_3_^2−^) (mM)	1.5 ± 0.1	0.3 ± 0.1
*K*_*i*_ (S_2_O_3_^2−^) (mM)	13 ± 1	7 ± 2
*k*_cat_/*K*_*m*_ (OAS) (M^−1^ s^−1^)	29 ± 12	(0.47 ± 0.06) × 10^5^
*k*_cat_/*K*_*m*_ (S_2_O_3_^2−^) (M^−1^ s^−1^)	193 ± 30	(9.5 ± 3.2) × 10^5^

*Note*: Values represent mean ± SEM from ≥3 independent measurements, obtained using at least two different protein batches.

We then investigated the ability of the two enzymes to utilize thiosulfate as an alternative sulfur donor with OAS. Kinetic analyses were performed under the same experimental conditions as above, replacing Na_2_S with Na_2_S_2_O_3_ (Figure 4e–h and Table 1). In contrast to the sulfide‐dependent reaction, no cooperativity toward OAS was observed for either enzyme (Hill coefficient *n* = 1), and the data were well described by the Michaelis–Menten model.

PaCysM was found to possess a much higher catalytic activity, with a *k*_cat_ of 286 ± 21 s^−1^, compared to only 0.29 ± 0.04 s^−1^ for PaCysK. Despite this large difference in turnover rates, both enzymes exhibited comparable substrate affinities for OAS (*K*_*m*_ of 6.1 ± 0.6 mM for PaCysM and 10 ± 4 mM for PaCysK) and thiosulfate (*K*_*m*_ of 0.3 ± 0.1 mM and 1.5 ± 0.1 mM, respectively). Consequently, PaCysM achieved a catalytic efficiency of ~0.47 × 10^5^ M^−1^ s^−1^ toward OAS, more than 1,500‐fold higher than that of PaCysK (~ 29 M^−1^ s^−1^). An even more pronounced difference was observed with thiosulfate, with PaCysM reaching a catalytic efficiency of 9.5 × 10^5^ M^−1^ s^−1^, nearly 5000‐fold greater than that of PaCysK (~193 M^−1^ s^−1^). These results underscore the strong functional specialization of PaCysM for thiosulfate utilization.

The two isoenzymes also exhibited marked differences in thermal stability. To assess thermal inactivation, the purified enzymes were incubated at increasing temperatures (37°C to 100°C) for 10 min, and residual activity was subsequently measured. The obtained T_50_ values, defined as the temperature at which 50% of the initial enzymatic activity is lost, were ~65°C for PaCysK and ~83°C for PaCysM (Figure S3). This difference parallels the distinct thermal stability profiles observed by CD thermal denaturation (Figure 2e), further confirming that PaCysM is more resistant to thermal denaturation.

### 2.3 Pre‐steady state kinetic studies

**FIGURE 5:**
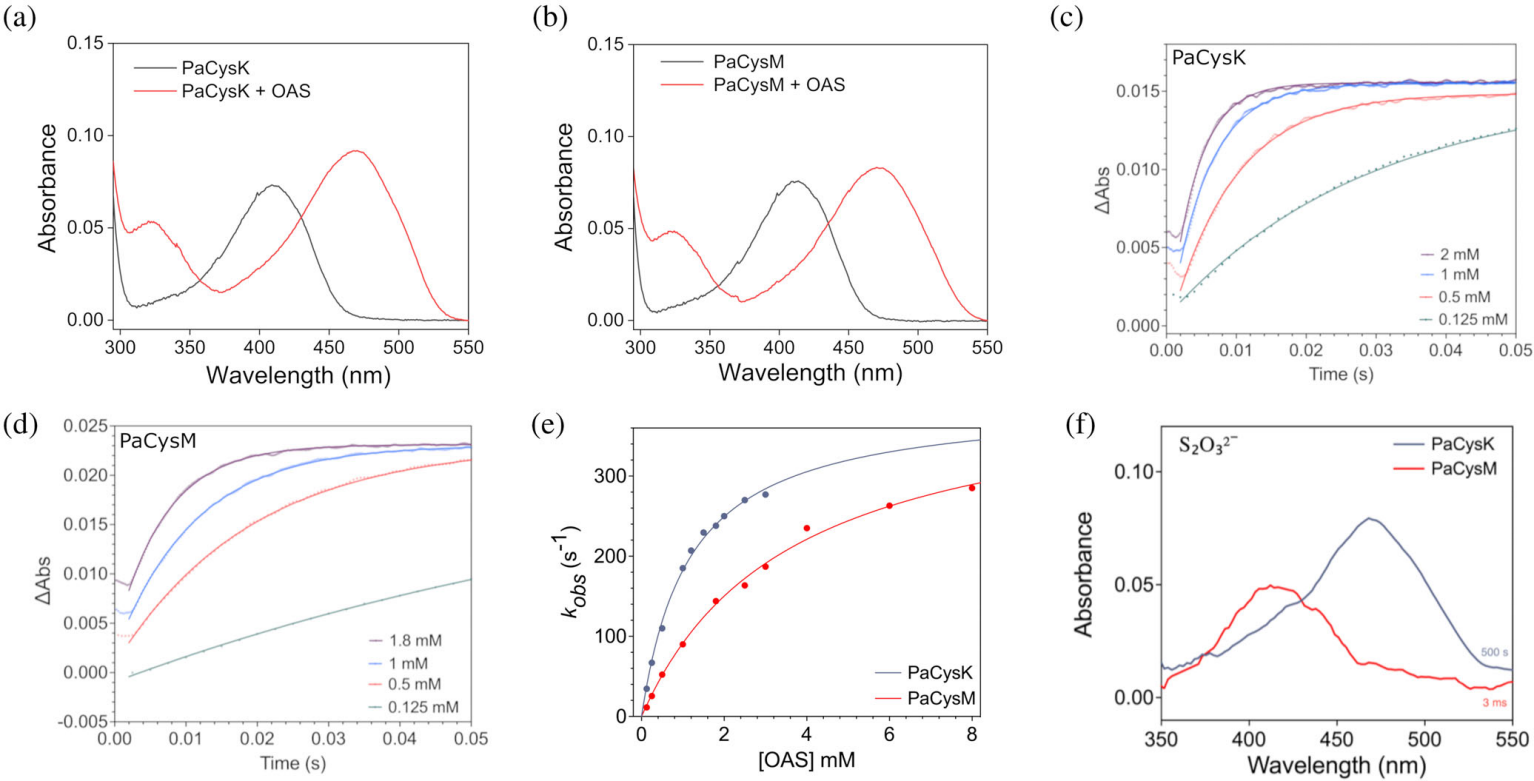
Pre‐steady‐state kinetics analysis. (a, b) UV‐visible absorption spectra of 15 μM PaCysK (a) and PaCysM (b) recorded before and after addition of 5 mM OAS. (c, d) Representative time courses for formation of the α‐aminoacrylate intermediate, acquired at 470 nm after stopped‐flow mixing 32 μM PaCysK (c) or PaCysM (d) with increasing concentrations of OAS. Solid lines represent the best fits to Equation (1) (Section 4). Light path = 0.2 cm. (e) Dependence on OAS concentration of the observed rate constants (*k*_obs_) measured for α‐aminoacrylate formation with PaCysK (gray) or PaCysM (red). Solid lines represent the best fits to Equation (2) (Section 4). (f) Absorption spectra acquired after stopped‐flow mixing 29 μM PaCysK (gray traces) or 29 μM PaCysM (red traces), both pre‐incubated with 40 mM OAS, with Na_2_S_2_O_3_ (10 mM for PaCysK or 6 mM for PaCysM). The depicted spectra for PaCysM and PaCysK were collected 3 ms and 500 s after mixing, respectively. While no reaction was detected for PaCysK even after 500 s, PaCysM completed the reaction within 3 ms, highlighting its markedly higher reactivity toward thiosulfate and broader substrate specificity.

PaCysK and PaCysM, upon reacting with OAS in the first half reaction of the ping‐pong mechanism, undergo the spectral changes shown in Figure 5a,b. Initially, both enzymes exhibit an absorption spectrum characteristic of their resting state, with a peak at ~412 nm arising from the internal Schiff base formed between the PLP cofactor and the active‐site lysine residue. Upon reaction with OAS, a red shift to ~470 nm is observed, consistent with the accumulation of the α‐aminoacrylate intermediate (Cook et al., 1992; Cook & Wedding, 1976; Tai et al., 1995). Concomitantly, an additional band appears at ~320 nm, likely reflecting the enolimine tautomer of this species. CD spectroscopy further supported the formation of the α‐aminoacrylate intermediate in both enzymes, showing a characteristic negative band in the 460–470 nm range (Figure S4), consistent with the absorbance data.

The kinetics of α‐aminoacrylate formation was investigated by stopped‐flow spectroscopy. Time‐resolved absorbance spectra confirmed the spectral transition observed in Figure 5a,b for the conversion of the internal Schiff base to the α‐aminoacrylate intermediate. Notably, immediately after OAS addition, a red shift of the peak at 412 nm to longer wavelengths (~418 nm) was detected, consistent with the formation of the external Schiff base (Tai et al., 2008). Spectra collected 12 and 24 ms after mixing reflected a combination of external Schiff base and α‐aminoacrylate species, in agreement with previous reports (Woehl et al., 1996) (Figure S5). Kinetic traces acquired at 470 nm after mixing increasing concentrations of OAS with PaCysK (Figure 5c) or PaCysM (Figure 5d) were fitted to a single‐exponential function. The observed rate constants (*k*_obs_) displayed a hyperbolic dependence on OAS concentration (Figure 5e) and were analyzed using Equation (2) (Section 4), according to (Woehl et al., 1996). The resulting kinetic parameters are summarized in Table 2. The *k*_max_ values, corresponding to the calculated *k*_obs_ at saturating OAS concentration and reporting on  the acetate β‐elimination step from the external OAS‐aldimine intermediate, leading to formation of the α‐aminoacrylate intermediate (Figure 1b), are comparable for PaCysK and PaCysM (394 ± 13 s^−1^ vs. 419 ± 17 s^−1^). In contrast, the apparent *c*_50_ value for OAS is higher for PaCysM (3.6 ± 0.3 mM) than for PaCysK (1.2 ± 0.1 mM), suggesting a lower affinity of OAS for PLP‐bound PaCysM compared to PaCysK.

**TABLE 2:** Kinetic parameters for the reaction of PaCysK and PaCysM with OAS.

	PaCysK	PaCysM
L‐OAS → α‐aminoacrylate + acetate		
*k*_max_	394 ± 13 s^−1^	419 ± 17 s^−1^
*c*_*50*_	1.2 ± 0.1 mM	3.6 ± 0.3 mM

The second half‐reaction, where a sulfur donor attacks the α‐aminoacrylate intermediate to form L‐cysteine/S‐sulfocysteine (Figure 1b) (Mozzarelli et al., 2011), was monitored by multi‐wavelength stopped‐flow spectrophotometry. Both Na_2_S and Na_2_S_2_O_3_ were tested as sulfur donors. After mixing OAS‐preincubated PaCysK or PaCysM with 1 mM Na_2_S, a rapid decay of the α‐aminoacrylate intermediate was observed, accompanied by the reappearance of the internal Schiff base, as indicated by the spectral shift from 460–470 to ~420 nm detected within 3 ms after mixing (data not shown). These results indicate that both enzymes react with Na_2_S with a second‐order rate *k* ≥ 2 × 10^6^ M^−1^ s^−1^. Consistently, CD spectra also show the disappearance of the α‐aminoacrylate signal upon addition of the sulfur donor, accompanied by the reappearance of the ~412 nm band (Figure S4). In contrast, when OAS‐preincubated PaCysK or PaCysM was rapidly mixed with Na_2_S_2_O_3_ (10 mM for PaCysK and 6 mM for PaCysM), the two enzymes displayed markedly distinct behaviors (Figure 5f). PaCysM fully regenerated the resting enzyme within 3 ms after mixing, consistent with a second‐order rate constant *k* ≥ 1.7 × 10^5^ M^−1^ s^−1^. In contrast, PaCysK retained the 470 nm absorption peak even at 500 s after mixing, indicating a much slower reactivity of the α‐aminoacrylate intermediate toward S_2_O_3_^2−^ compared to PaCysM.

These observations indicate that, while both enzymes utilize sulfide as sulfur donor with comparable efficiency, PaCysM exhibits markedly higher reactivity toward thiosulfate,   in line with the broader substrate specificity observed under steady‐state conditions.

### 2.4 Analysis of *P. aeruginosa* mutants lacking *cysK* and/or *cysM*

To investigate the activity of PaCysK and PaCysM in *P. aeruginosa*, the *cysK* (PA2709) and *cysM* (PA0932) genes were independently deleted from the chromosome of the *P. aeruginosa* model strain PAO1 *via* allelic exchange to obtain the ∆*cysK* and ∆*cysM* mutants, respectively. Additionally, a ∆*cysKM* double mutant lacking both *cysK* and *cysM* was constructed using a stepwise procedure (Table S2). The resulting strains were assessed for their ability to grow in M9 minimal medium supplemented with glucose as the sole carbon source. This medium lacks cysteine and contains MgSO_4_ as the sole sulfur source, which bacteria convert to inorganic sulfide through the sulfate reduction pathway (Guédon & Martin‐Verstraete, 2007).

**FIGURE 6:**
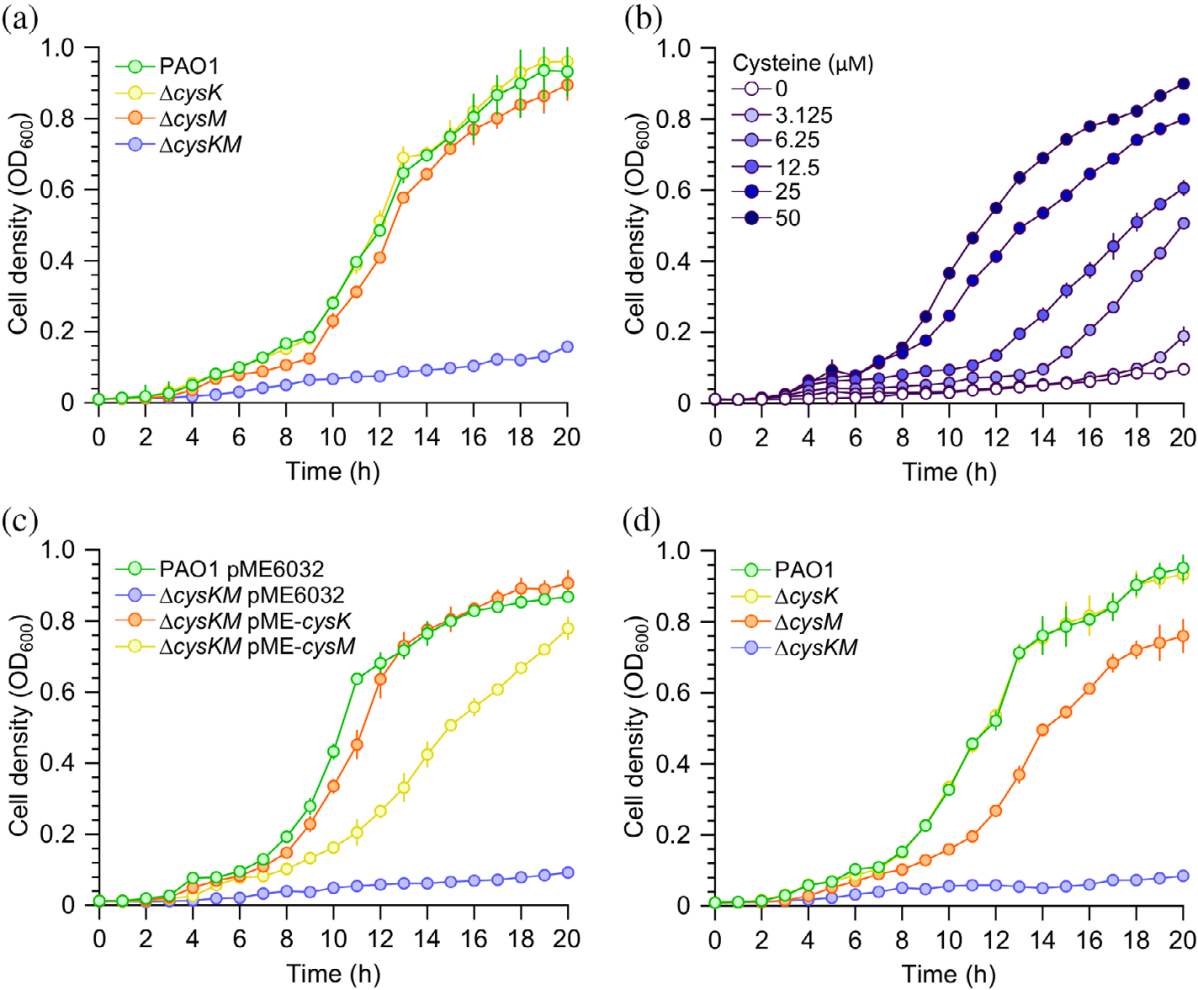
Growth curves of *P. aeruginosa* PAO1 wild type and mutants. (a) Growth curves of *P. aeruginosa* PAO1 wild type and the indicated isogenic mutants in the M9 minimal medium, in which MgSO_4_ is the sole sulfur source. (b) Growth curves of the ∆*cysKM* double mutant in the M9 minimal medium supplemented with cysteine at the indicated concentrations. (c) Growth curves of *P. aeruginosa* PAO1 wild type carrying the pME6032 empty vector and of the Δ*cysKM* double mutant alternatively carrying the pME6032 empty vector or the pME6032‐derived plasmids for IPTG‐inducible expression of *cysK* (pME‐*cysK*) or *cysM* (pME‐*cysM*). The strains were cultured in the M9 minimal medium, in which MgSO_4_ is the sole sulfur source, supplemented with 0.5 mM IPTG. (d) Growth curves of *P. aeruginosa* PAO1 wild type and the indicated isogenic mutants in a modified M9 minimal medium in which thiosulfate is the only sulfur source. Mean values and standard deviations derive from three independent experiments.

As shown in Figure 6a, the ∆*cysK* and ∆*cysM* mutants exhibited growth rates comparable to that of the parental strain, whereas the growth of the ∆*cysKM* double mutant was almost completely abolished. The minimal and delayed growth of the ∆*cysKM* strain in the absence of cysteine may be attributed to additional low‐activity OASS enzymes encoded in the *P. aeruginosa* PAO1 genome or to cysteine production *via* the reverse transsulfuration pathway. Supplementation of M9 medium with increasing concentrations of cysteine restored ∆*cysKM* growth in a dose‐dependent manner (Figure 6b). Moreover, the growth defect of the ∆*cysKM* mutant could be rescued by ectopic expression of either *cysK* or *cysM* *via* the pME6032‐derived plasmids pME‐*cysK* and pME‐*cysM*. These findings confirm that PaCysK and PaCysM play a redundant yet primary role in cysteine biosynthesis in *P. aeruginosa*. Interestingly, under equivalent IPTG induction conditions, presumably resulting in comparable gene expression levels, expression of *cysK* fully complemented the growth defect of the ∆*cysKM* mutant in M9 medium with MgSO_4_ as the sole sulfur source, whereas expression of *cysM* only partially restored growth under the same conditions (Figure 6c), in line with the higher activity of purified PaCysK in the presence of sulfide.

Impaired growth was also observed when the ∆*cysKM* strain was cultured in a modified M9 medium in which sulfate was replaced by thiosulfate. In this condition, deletion of *cysM* decreased *P. aeruginosa* growth, whereas the ∆*cysK* mutant exhibited a growth rate comparable to the parental strain (Figure 6d), confirming that thiosulfate is preferentially utilized by PaCysM for cysteine biosynthesis. Overall, these results demonstrate that PaCysK and PaCysM are both required and functionally redundant for cysteine biosynthesis from either sulfide (derived from MgSO_4_) or thiosulfate in *P. aeruginosa* under cysteine‐ limiting conditions. Our findings are consistent with the biochemical data showing that PaCysK exhibits higher catalytic activity in the presence of inorganic sulfide compared to PaCysM, while PaCysM is more efficient in utilizing thiosulfate.

### 2.5 Comparative structural analysis of PaCysK and PaCysM reveals determinants of substrate specificity

To investigate the molecular basis for the distinct substrate preferences of *P. aeruginosa* enzymes, we performed a comparative structural analysis of PaCysK and PaCysM. In the absence of experimentally determined structures, we used AlphaFold‐predicted models (Jumper et al., 2021) (AF‐Q9I0D3‐F1 for PaCysK and AF‐Q9I526‐F1 for PaCysM). Both models display high confidence (pLDDT >90) (Figure S6) and represent the open (substrate‐free) conformation, consistent with the known catalytic cycle of OASS enzymes, which alternate between open and closed states during catalysis (Hicks et al., 2022).

**FIGURE 7:**
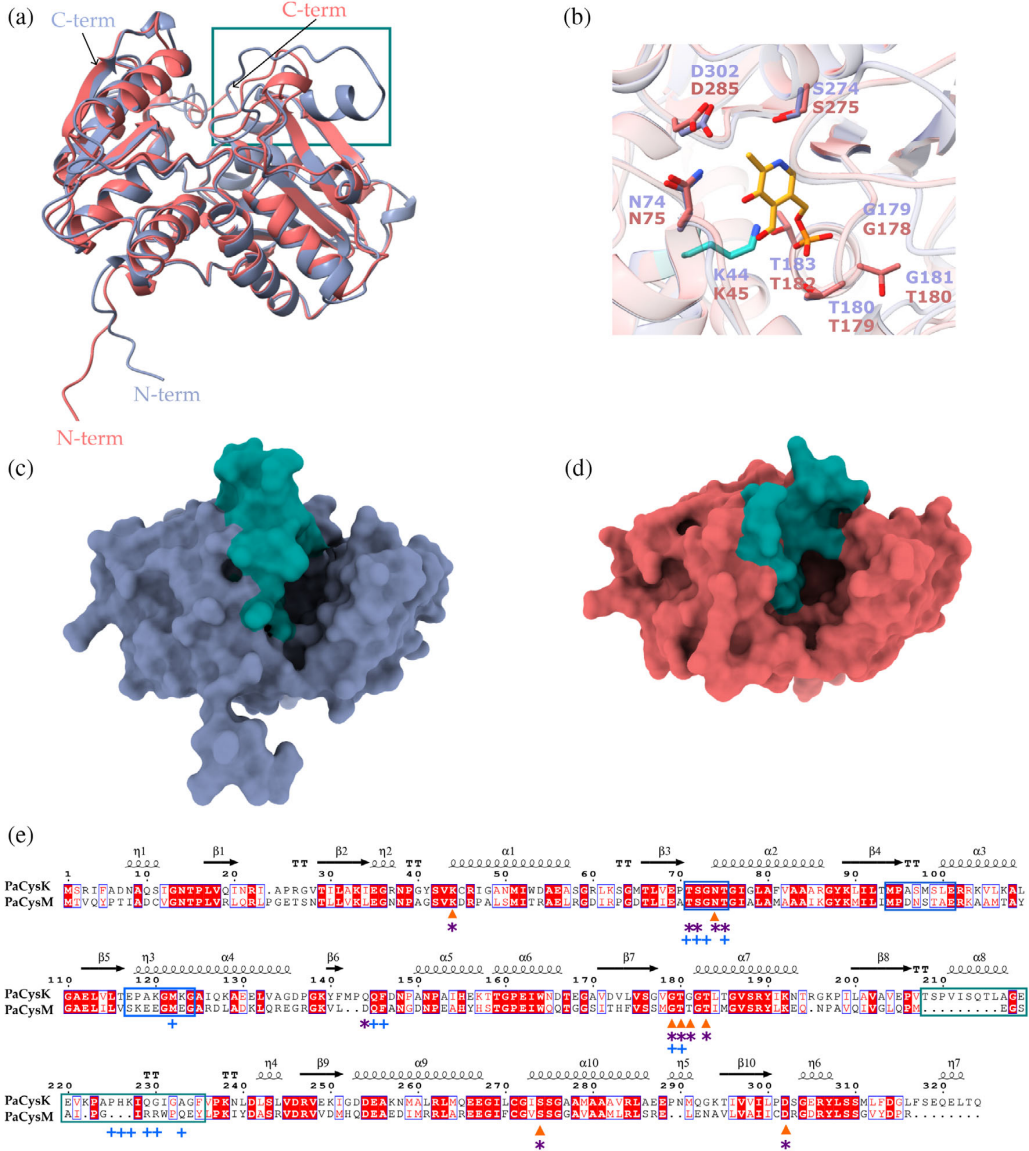
Structural analysis of PaCysK and PaCysM. (a) Structure‐based superposition of AlphaFold‐predicted models of PaCysK (gray, AF‐Q9I0D3‐F1) and PaCysM (red, AF‐Q9I526‐F1). (b) Close‐up view of the active sites of PaCysK and PaCysM with the PLP shown in yellow. The PLP was extracted from the MtCysK1 holoenzyme structure (PDB ID: 2Q3D; Schnell et al., 2007), which was structurally aligned to the models of PaCysK and PaCysM. Interacting residues are shown as sticks and numbered according to the *P. aeruginosa* enzymes; the lysine involved in Schiff base formation is highlighted in cyan. (c, d) Surface representations of the active site cavities of PaCysK (c) and PaCysM (d), illustrating the differing degrees of openness between the two enzymes. (e) Structure‐based sequence alignment of PaCysK and PaCysM. Identical residues are shown on a red background; conserved substitutions are shown in red on a white background. Residues putatively involved in PLP or α‐aminoacrylate binding, based on structural superposition with MtCysK1 complexes (PDB ID: 2Q3B and 2Q3D), are indicated with orange triangles and purple asterisks, respectively. Residues potentially involved in Salmonella peptide binding (see below) are marked with blue crosses. The region spanning residues 208–235 (see text) is highlighted with a green box.

The two enzymes share the fold type II of PLP‐dependent OASS, with an RMSD of 0.7 Å between 255 pruned atom pairs (3.5 Å across all 298 pairs), indicating a high degree of structural similarity (Figure 7a). Each monomer consists of two α/β domains: a small N‐terminal domain with a four‐stranded β‐sheet flanked by four α‐helices, and a larger C‐terminal domain comprising a six‐stranded β‐sheet flanked by four α‐helices. The PLP cofactor binds at the interdomain cleft, forming an internal aldimine with a conserved lysine (K44 in PaCysK), as observed in other OASS enzymes (Chattopadhyay et al., 2007).

The AlphaFold models of PaCysK and PaCysM were superimposed onto the open‐state crystal structure of *M. tuberculosis* CysK1 (MtCysK1), solved in complex with PLP (PDB ID: 2Q3B; (Schnell et al., 2007)). Since MtCysK1 has also been solved in the closed, α‐aminoacrylate‐bound state (PDB ID: 2Q3D; (Schnell et al., 2007)), we used this pair of structures to map onto PaCysK and PaCysM the regions expected to undergo the open‐to‐closed transition. The major rearrangements in PaCysK involve the loop containing the conserved 71‐TSGNT‐75 motif (72‐TSGNT‐76 in PaCysM), the 94‐MPETMSLE‐101 loop (95‐MPDNSTAE‐102 in PaCysM), and the 117‐EPAKGMKG‐124 segment (118‐SKEEGMEGA‐125 in PaCysM), which in the MtCysK1‐α‐aminoacrylate complex folds over the active site (Schnell et al., 2007).

The PLP‐binding residues (PaCysK numbering: K44, N74, G179, T180, G181, T183, S274, D302) are largely conserved in PaCysK and PaCysM, supporting proper cofactor coordination (Figure 7b). Similarly, the residues involved in α‐aminoacrylate binding, identified through structural superposition of the PaCys models onto the MtCysK1‐aminoacrylate complex (PDB ID: 2Q3D), also show a high degree of conservation (PaCysK numbering: K44, T71, S72, N74, T75, Q145, G179, T180, G181, T183, S274, D302) (Figure 7e), consistent with a shared catalytic mechanism.

However, while the active site cleft is similar in the two enzymes (i.e., PLP binding region is conserved) notable differences emerge in the regions surrounding the catalytic pocket. Variations in this region have been shown to influence substrate access and specificity (Chattopadhyay et al., 2007; Claus et al., 2005; Hicks et al., 2022). Specifically, in *P. aeruginosa*, the segment spanning residues 229–235 in PaCysK adopts a compact QGIGAGF motif, which contributes to a narrower and more linear active‐site cleft. In contrast, the corresponding region in PaCysM (residues 214–220) features a bulkier and more solvent‐exposed RRWPQEY motif, resulting in a more accessible catalytic pocket. In addition, the adjacent loop (residues 208–228 in PaCysK) is longer and more extended compared to the equivalent, shorter and more recessed loop in PaCysM. These structural differences likely restrict sulfur donor access in PaCysK, creating a tunnel facing the active site, whereas the more open architecture is present in PaCysM (Figure 7c,d). This difference is expected to become even more pronounced in the closed conformations of the enzymes, which are likely stabilized upon binding aminocarboxylate substrates (Figure S7). Beyond steric factors, differences in electrostatic surface potential also likely play a role. Entrance to the sulfur donor site in PaCysM displays a more positively charged environment compared to PaCysK (Figure S7), which may further enhance its interaction with negatively charged sulfur donors. Notably, this charge enrichment is partly due to residue R214, which is uniquely present in PaCysM.

### 2.6 Peptide binding to PaCysK

The interaction between CysE and CysK involves insertion of the C‐terminal peptide of CysE into the active site of CysK, forming the CSC and inhibiting CysK activity to regulate cysteine biosynthesis (Kant et al., 2019; Salsi et al., 2010; Spyrakis et al., 2013). A strictly conserved C‐terminal isoleucine is essential for binding, contributing ~80% of the interaction energy and anchoring the peptide within the active site (Salsi et al., 2010). Notably, its deletion or substitution abolishes binding to CysK (Campanini et al., 2005; Mino et al., 1999).

**FIGURE 8:**
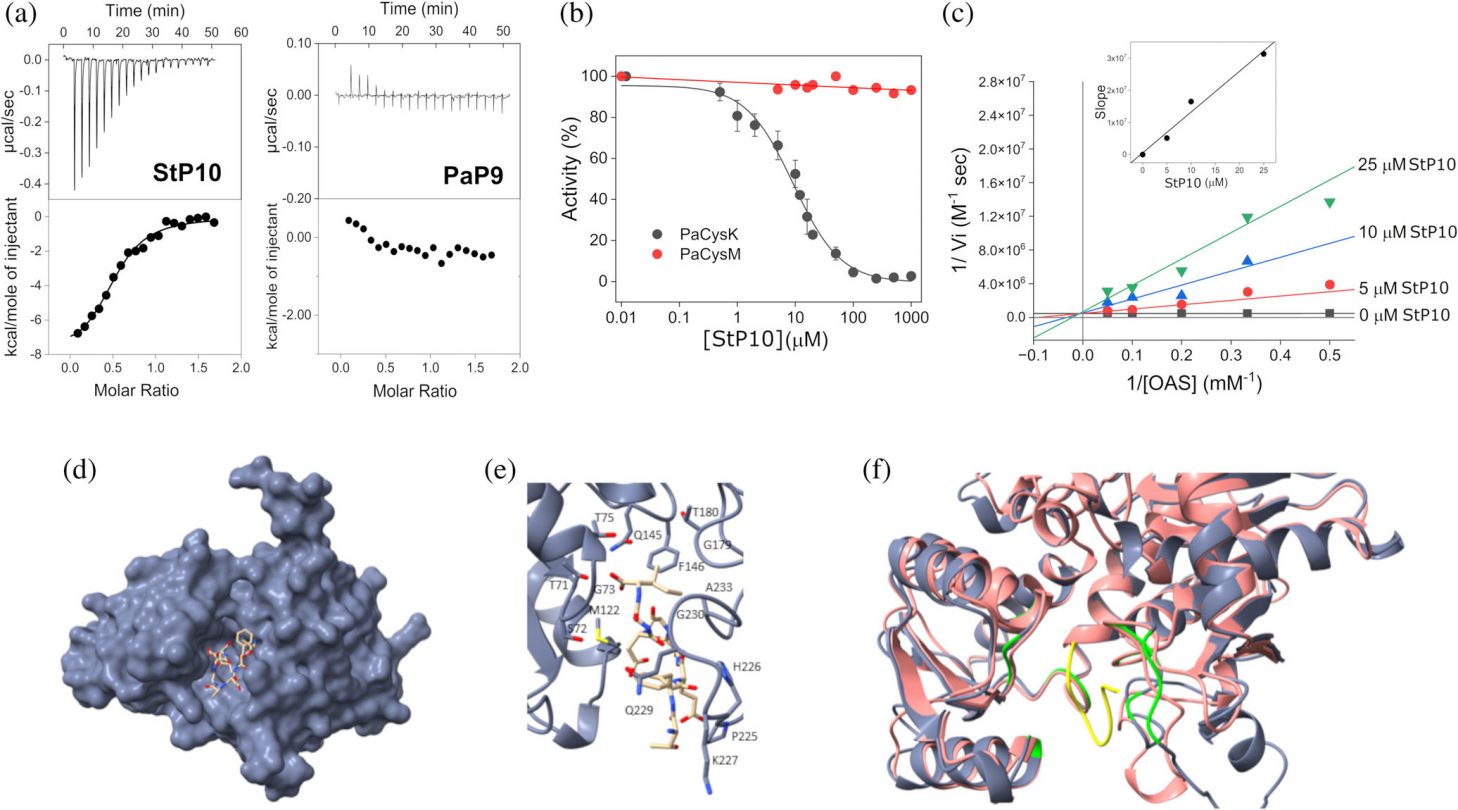
Peptide binding to PaCysK. (a) Representative ITC thermograms (top panel) and the derived binding isotherms (bottom panel) of PaCysK titration with the StP10 (corresponding to the C‐terminus of StCysE) and PaP9 (corresponding to the C‐terminus of PaCysE1) peptides at 25°C. (b) Dose–response curves of PaCysK and PaCysM percentage activity plotted against StP10 peptide concentration. Initial rates were determined in the presence of increasing peptide concentrations (0–1000 μM) with fixed 10 mM OAS and 0.5 mM Na_2_S for PaCysK or 5 mM Na_2_S for PaCysM at 37°C. (c) Double‐reciprocal (Lineweaver–Burk) plots of initial velocity versus OAS concentration, indicating competitive inhibition. Reactions were performed in the presence of increasing StP10 concentrations (0–25 μM). The inset shows the secondary plot of slope values versus peptide concentration. (d) Surface representation of PaCysK with the peptide derived from the crystal structure of *S. enterica* serovar Typhimurium (PDB ID: 4LI3). (e) Close‐up view of the putative interaction region between PaCysK and the peptide, obtained by structural superposition of the PaCysK model with the 4IL3 structure. (f) Structural superposition of the PaCysK model (gray) with the PaCysM model (pink). The Salmonella peptide (from 4LI3) is shown in yellow. Regions on PaCysK putatively involved in peptide binding are highlighted in green.

Structural studies of CysK in complex with CysE‐derived or model peptides have elucidated the molecular basis of CSC assembly, providing insight into the CysK–CysE interaction in the absence of atomic‐resolution structures of the full complex (Francois et al., 2006; Huang et al., 2005; Salsi et al., 2010; Schnell et al., 2007; Schnell et al., 2015). In *P. aeruginosa*, two isoforms of CysE are present (PaCysE1 and PaCysE2, ~ 36% sequence identity), which share moderate sequence similarity with CysE of *Salmonella enterica* serovar Typhimurium (StCysE) (approximately 40% for PaCysE1 and 31% for PaCysE2) (Figure S8). Notably, both Pseudomonas CysE isoenzymes lack the invariant C‐terminal isoleucine. However, PaCysK retains all canonical residues required for CysE‐peptide interaction (T71, S72, T75, Q145, PaCysK numbering) (Campanini et al., 2015; Francois et al., 2006; Huang et al., 2005; Salsi et al., 2010; Schnell et al., 2007; Schnell et al., 2015), suggesting it could still recognize a canonical CysE‐derived motif. To test this hypothesis, we synthesized two peptides: one corresponding to the C‐terminal region of PaCysE1 (KDEDGNPAA, hereafter referred to as PaP9) and a decapeptide derived from the C‐terminus of StCysE (HHTFEYGDGI, hereafter referred to as StP10). The StP10 peptide encompasses the well‐characterized interaction motif responsible for binding CysK in *S. enterica* and is therefore commonly used as a model peptide for CSC studies. Isothermal titration calorimetry (ITC) experiments revealed that PaP9 does not bind PaCysK, whereas StP10 binds PaCysK with an exothermic profile that fits a single‐site binding model, yielding a dissociation constant (*K*_*d*_) of 8 ± 2 μM (ΔH = −10.7 ± 1.3 kcal mol^−1^) (Figure 8a). Given its ability to bind PaCysK, StP10 was used to assess the functional consequences of this interaction. StP10 inhibited PaCysK with an IC_50_ of 9.2 ± 0.8 μM (Figure 8b). In contrast, no inhibition was observed for PaCysM. Lineweaver‐Burk analysis showed that StP10 acts as a competitive inhibitor of PaCysK, with a *Ki* of ~0.5 μM (Figure 8c). These data support the idea that the lack of the conserved isoleucine and additional key residues in the C‐terminal region of PaCysE1 prevents CSC formation in *P. aeruginosa*.

We next performed a structural analysis of the PaCysK–peptide interaction. As a reference, we used the crystal structure of *H. influenzae* CysK (HiCysK) in complex with the C‐terminal peptide of StCysE (PDB ID: 4LI3, unpublished), which is two residues shorter than the peptide employed in our biochemical assays. This structure was superimposed onto the AlphaFold model of PaCysK. The two structures superimposed well (RMSD between 304 pruned atom pairs = 0.6 angstroms Å; across all 311 pairs: 0.8 Å), indicating no major conformational changes upon peptide binding. The peptide binds into the PaCysK active‐site pocket (Figure 8d). Key residues mediating peptide binding in HiCysK, as identified by pdbsum (https://www.ebi.ac.uk/thornton-srv/databases/pdbsum) of 4LI3, are conserved in PaCysK (T71, S72, G73, T75, M122, Q145, F146, G179, T180, P225, H226, K227, Q229, G230, and A233, PaCysK numbering; Figures 7e and 8e). This confirms that PaCysK retains a structurally competent binding pocket capable of accommodating the CysE peptide with a geometry likely resembling that observed in the HiCysK–peptide complex.

In contrast, structural comparison with PaCysM revealed significant differences at the peptide‐binding interface. Although nine of the binding‐site residues are conserved, including the TSGNT motif, several critical substitutions are present. For instance, Q229 in PaCysK is replaced by R214 in PaCysM; G230 and A233 are substituted with R215 and Q218, respectively. Additionally, the loop spanning residues 208–227 in PaCysK is shorter in PaCysM (Figure 7e), resulting in a more open active‐site region that fails to establish multiple key contacts with the peptide (Figure 8f).

## 3 DISCUSSION

Cysteine biosynthesis is a central metabolic process in bacteria, closely linked to redox homeostasis, stress resistance, and virulence. In some species, this pathway is supported by the coexistence of two OASS isoenzymes, CysK (OASS‐A) and CysM (OASS‐B). Although these enzymes share a conserved PLP‐dependent catalytic mechanism and significant sequence similarity, comparative studies across diverse bacterial species have revealed marked differences in substrate specificity, regulation, and physiological relevance, suggesting that they fulfill non‐equivalent roles *in vivo* rather than acting as simple functional backups. Dissecting the mechanistic basis of this divergence within a single organism is therefore essential to understand how redundancy and specialization are balanced at the molecular level.

In this study, we provide a comprehensive functional and mechanistic comparison of the two OASS enzymes PaCysK and PaCysM from *P. aeruginosa*, revealing overlapping yet clearly specialized roles in cysteine metabolism, that likely contribute to sulfur metabolism flexibility in this pathogen.

Steady‐state kinetic analyses showed that both PaCysK and PaCysM follow the conserved ping‐pong mechanism for the canonical OAS‐dependent reaction, but differ in their preference for the sulfur donor in the second half‐reaction. PaCysK is preferentially optimized for sulfide‐dependent cysteine biosynthesis, displaying a higher catalytic efficiency toward sulfide (*k*_cat_/*K*_*m*_ ≈1 × 10^7^ M^−1^ s^−1^) compared to PaCysM (≈1.5 × 10^5^ M^−1^ s^−1^). These values are consistent with those reported for other bacterial CysK enzymes and are comparable to those of *S. enterica* serovar Typhimurium (≈10^6^–10^7^ M^−1^ s^−1^), although lower than that of the highly efficient *E. coli* CysK, which displays a high turnover number (*k*_cat_ = 2030 s^−1^, *K*_*m*_ (Na_2_S) ~ 6 μM; Kaushik et al., 2021; Mino et al., 2000; Tai et al., 1993). In contrast, PaCysM exhibits a pronounced preference for thiosulfate as nucleophile in the second half‐reaction. Its catalytic efficiency toward thiosulfate (*k*_cat_/*K*_*m*_ ≈9.5 × 10^5^ M^−1^ s^−1^) is substantially higher than that of PaCysK (*k*_cat_/*K*_*m*_ ≈1.9 × 10^2^ M^−1^ s^−1^) and closely matches that of *E. coli* CysM (≈10^6^ M^−1^ s^−1^; Zhao, Kumada, et al., 2006), underscoring the specialization of PaCysM as a thiosulfate‐adapted isoform. Qualitative LC–MS/MS product analyses independently corroborate this functional distinction. When sulfide was used as the sulfur donor, both enzymes produced cysteine with consumption of OAS. In contrast, under thiosulfate‐supplemented conditions, PaCysM generated a prominent S‐sulfocysteine signal accompanied by full OAS consumption, whereas PaCysK displayed only limited S‐sulfocysteine formation and substantial residual unreacted OAS.

Stopped‐flow analysis of the pre‐steady‐state kinetics supports the view that the mechanistic differences between PaCysK and PaCysM primarily arise during the sulfur incorporation step, rather than from differences in OAS binding or α‐aminoacrylate formation. Both PaCysK and PaCysM form the α‐aminoacrylate intermediate at similar maximal rates (*k*_max_). These β‐elimination rates are comparable to those reported for *S. enterica* serovar Typhimurium CysK (*k*_max_ = 300 s^−1^; Tai et al., 2008; Woehl et al., 1996), but significantly higher than that of *M. tuberculosis* CysM (*k*_max_ = 0.025 s^−1^; O'Leary et al., 2008), despite a comparable *c*_50_ value (5 mM). A key mechanistic distinction between the two isoforms emerges during sulfur donor utilization. While both enzymes efficiently complete catalysis in the presence of sulfide, only PaCysM reacts rapidly with thiosulfate, regenerating the internal aldimine within the instrument dead time. In contrast, PaCysK remains trapped in the α‐aminoacrylate intermediate for several hundred seconds, consistent with a markedly slower nucleophilic attack.

These data suggest that PaCysK corresponds to a canonical OASS‐A enzyme, functioning predominantly as a sulfide‐dependent cysteine synthase, whereas PaCysM displays the properties of an OASS‐B/S‐sulfocysteine synthase, efficiently catalyzing thiosulfate‐dependent sulfur incorporation to form S‐sulfocysteine. This classification is consistent with functional assignments reported for OASS isoforms in other bacterial systems (Bermúdez et al., 2010; Hensel & Trüper, 1983; Nakamura et al., 1984).

Structural analysis of the AlphaFold models of PaCysK and PaCysM provides a structural framework that rationalizes the distinct substrate preferences observed at the kinetic level. Despite belonging to the same family of PLP‐dependent OASSs and sharing a conserved catalytic core, the two enzymes display architectural differences that are well positioned to modulate sulfur donor accessibility and selectivity. OASS‐A and OASS‐B enzymes have been structurally characterized from a range of bacterial species, enabling comparison of the two isoenzymes and providing insight into active‐site conformation and isoform‐specific activity. The AlphaFold models of PaCysK and PaCysM closely resemble the canonical structures of OASS‐A and OASS‐B, respectively, and retain the conserved active‐site lysine required for PLP binding and catalysis (Bettati et al., 2000; Raj et al., 2012; Schnell et al., 2007). The strong conservation of key catalytic residues in PaCysK and PaCysM supports a shared catalytic mechanism, indicating that functional divergence is unlikely to arise from differences in chemistry *per se*. Rather, it appears to stem from differences in active‐site architecture and electrostatic environment.

Comparative structural analysis suggests that in PaCysK structural elements appear to restrict access of sulfur donors by shaping a relatively narrow tunnel leading to the active site. Such an arrangement is consistent with preferential utilization of the small and highly reactive sulfide nucleophile. In contrast, PaCysM displays a more open architecture, which may facilitate accommodation of bulkier or alternative sulfur donors, in agreement with previous structural and functional analyses of OASS‐B enzymes (Hicks et al., 2022). Consistently, electrostatic surface analysis reveals a more positively charged active‐site region in PaCysM compared to PaCysK (Figure S7). The presence of an arginine residue characteristic of CysM enzymes (R214 in PaCysM) contributes to this positively charged environment and has been proposed to play a role in thiosulfate binding (Bettati et al., 2000).

It is also worth noting that OASS enzymes typically undergo an open‐to‐closed conformational change upon OAS binding, as observed in the structure of the MtCysK1‐aminoacrylate complex (Hicks et al., 2022; Schnell et al., 2015). Both PaCysK and PaCysM are therefore likely to undergo a similar transition during catalysis, and the structural differences observed in the open conformation captured by our AlphaFold model may become even more pronounced in the closed state. This dynamic aspect may further amplify substrate selectivity by differentially shaping the catalytic pocket during turnover.

Importantly, the structural and kinetic distinctions between PaCysK and PaCysM are mirrored by their physiological roles *in vivo*. A Δ*cysM* mutant of *P. aeruginosa* exhibits reduced growth rate when thiosulfate is supplied as the sole sulfur source, whereas a Δ*cysK* strain grows comparably to the wild type under the same conditions. This phenotype indicates that PaCysM is the main OASS supporting thiosulfate assimilation *in vivo*. In its absence, thiosulfate is converted to cysteine with lower efficiency, likely reflecting the limited catalytic capacity of PaCysK for this substrate, in accordance with biochemical data. Conversely, when sulfate (e.g., MgSO_4_) is provided as the sulfur source, both Δ*cysK* and Δ*cysM* strains grow similarly to the parental strain. As sulfate is metabolized through the assimilatory reduction pathway to sulfide, which is then incorporated into cysteine by OASS, this result suggests that either isoenzyme can fulfill this function when sulfide is available. However, experiments performed in genetic backgrounds that alternatively express *cysK* or *cysM* at similar levels indicate that PaCysK is more active than PaCysM in producing cysteine in the presence of MgSO_4_, implying that PaCysK likely supports most of the cysteine biosynthesis from sulfide in *P. aeruginosa*, whereas PaCysM plays a specialized adaptive role. A similar pattern has been observed in *S. enterica* serovar Typhimurium, where CysM is essential for growth on thiosulfate, whereas CysK‐deficient strains show no growth defect under this condition, underscoring the prominent role of CysM in thiosulfate‐dependent cysteine biosynthesis via S‐sulfocysteine formation (Nakamura et al., 1983).

Therefore, PaCysK and PaCysM are not functionally equivalent enzymes operating in parallel, but rather are tuned to distinct sulfur regimes. PaCysK appears optimized for efficient cysteine production under sulfide‐rich conditions, whereas PaCysM likely supports cysteine biosynthesis when sulfide availability is limited or alternative sulfur sources prevail. The greater thermal stability of PaCysM, revealed by both thermal denaturation and inactivation assays, further supports this model, suggesting structural adaptations that may preserve enzymatic function under stress conditions frequently encountered during infection or nutrient limitation. Such specialization within a partially redundant enzymatic system likely represents an evolutionary strategy to ensure robust cysteine biosynthesis across diverse environmental niches.

Beyond catalysis, CysK enzymes are commonly regulated via interaction with CysE, the serine acetyltransferase responsible for OAS production. In many bacteria, these enzymes form the CSC, in which the C‐terminal tail of CysE inserts into the active site of CysK, inhibiting its activity. Recently, a “competitive‐allosteric” mechanism through which CysK is able to selectively recruit the substrate even when bound to its natural inhibitor has been proposed (Kaushik et al., 2021). This interaction critically depends on a conserved C‐terminal isoleucine residue in CysE, which provides most of the binding energy required for complex formation (Campanini et al., 2005; Marchetti et al., 2021; Mino et al., 1999; Salsi et al., 2010; Salsi, Campanini, et al., 2010). In *P. aeruginosa*, however, both annotated CysE isoforms (PaCysE1 and PaCysE2) lack this conserved isoleucine, suggesting that the canonical CSC is absent or non‐functional in this organism, in analogy to what has been reported for *B. subtilis* and *N. gonorrhoeae* (McGarvie et al., 2025; Tanous et al., 2008). In line with this interpretation, our peptide‐binding experiments show that the last nine C‐terminal amino acids of PaCysE1 do not interact detectably with PaCysK under the conditions tested. At the same time, our data indicate that PaCysK retains the ability to recognize a canonical CysE‐like motif. This is evidenced by its interaction with a peptide derived from the C‐terminal region of *S. enterica* serovar Typhimurium CysE, which contains the conserved isoleucine and effectively inhibits PaCysK. This finding suggests that the PaCysK active site remains competent for CSC‐like interactions, even though the native *P. aeruginosa* CysE proteins do not appear to exploit this regulatory mechanism. In contrast, PaCysM showed no inhibition by the same peptide, consistent with its classification as an OASS‐B enzyme, which typically does not participate in CSC formation.

Structural comparison using the crystal structure of HiCysK in complex with Salmonella peptide (PDB: 4LI3) as a reference revealed that the key residues involved in peptide binding are conserved in PaCysK, but largely absent in PaCysM. Notably, the loop spanning residues 208–227 in PaCysK is shorter in PaCysM, thus failing to establish the network of contacts required for stable peptide binding. These structural differences may account for the inability of PaCysM to bind the peptide and underscore the role of upstream regions in mediating this interaction. Consistent with this, previous work has shown that full‐length *E. coli* CysE is a much more potent inhibitor of CysK than the isolated C‐terminal decapeptide, with approximately 250‐fold higher affinity (Huang et al., 2005), highlighting the importance of extended interface contacts for stable complex formation.

Taken together, these observations suggest that, although PaCysK retains the structural features necessary for peptide‐mediated regulation, *P. aeruginosa* has likely diverged from CSC‐dependent control of cysteine biosynthesis. The loss of the C‐terminal isoleucine in PaCysE isoforms, along with the low overall sequence similarity to *E. coli* CysE, points to a divergent regulatory strategy. Rather than relying on post‐translational control via CSC formation, *P. aeruginosa* may control cysteine biosynthesis primarily through transcriptional regulation (e.g., *via* the LysR‐type regulator CysB) and direct feedback inhibition of CysE.

Notably, C‐terminal peptide motifs containing an isoleucine residue have also been found in several non‐CysE proteins known to interact with CysK homologs, including the CdiA^Ec5396^ toxin from *E. coli* (Johnson et al., 2016), the oxygen‐sensitive prolyl hydroxylase (EGL‐9) from *Caenorhabditis elegans*, which binds a CysK homolog (Ma et al., 2012), and the transcription factor CymR in *B. subtilis* (Tanous et al., 2008). Structural studies have shown that the CdiA^Ec5396^ toxin exploits CSC‐like interactions to engage CysK, inserting its C‐terminal GYGI motif into the active site of CysK and anchoring the complex (Johnson et al., 2016).

In this context, the retention of peptide‐binding features in PaCysK suggests that this enzyme may interact with alternative regulatory partners in *P. aeruginosa*, as observed for CymR in *B. subtilis* (Tanous et al., 2008), and is consistent with the proposed moonlighting role of CysK (Campanini et al., 2015). This raises the intriguing possibility that PaCysK plays a broader regulatory role in alternative cellular processes, a hypothesis that warrants further investigation.

These findings also have important therapeutic implications. CysK and CysM are absent in humans, which further highlights their pharmacological attractiveness and reduces the likelihood of host‐associated toxicity. Although these enzymes have already been proposed as druggable nodes in other pathogens (Brunner et al., 2016; Joshi et al., 2019; Poyraz et al., 2013), *P. aeruginosa* appears to deviate from the canonical CSC‐mediated regulation described in bacteria such as *E. coli* and *H. influenzae*, implying alternative and yet unexplored modes of control. The coexistence of two isoforms further implies that effective inhibition of cysteine biosynthesis may require targeting both activities.

Overall, our results indicate that PaCysK and PaCysM are unlikely to operate as isolated catalytic units but may engage in additional, as yet unidentified, regulatory interactions. Mapping such protein networks and defining the molecular logic of partner recognition would not only clarify the architecture of cysteine metabolism in *P. aeruginosa* but also reveal new layers of pharmacological vulnerability, potentially extending therapeutic strategies beyond the active site to interface regions. Future studies should therefore focus on identifying these regulatory partners and defining their structural and functional interplay with PaCysK and PaCysM.

## 4 MATERIALS AND METHODS

### 4.1 Chemicals and materials

All standard chemical reagents were purchased from Sigma‐Aldrich (St. Louis, MA), Thermo Fisher Scientific (Waltham, MA), Honeywell (Charlotte, NC), unless stated otherwise, and used as supplied. The StP10 (HHTFEYGDGI) and PaP9 (KDEDGNPAA) peptides were purchased from GenScript (Piscataway, NJ).

### 4.2 Recombinant protein production

The pET28a^+^ vectors containing the gene sequences for PaCysK (UniProt ID: Q9I0D3) and PaCysM (UniProt ID: Q9I526), each featuring an N‐terminal 6x‐His tag, were obtained from Genscript. The constructs were transformed into *E. coli* Rosetta (DE3) expression host cells (Novagen, Madison, WI) for protein expression. Cultures were grown in Luria–Bertani (LB) medium at 37°C until an optical density at 600 nm of 0.6 was reached. Protein expression was induced with 0.5 mM isopropyl‐β‐D‐1‐thiogalactopyranoside (IPTG), followed by incubation for 16 h at 24°C. Cells were harvested by centrifugation and resuspended in lysis buffer composed of 20 mM NaP, 150 mM NaCl, 10 mM imidazole, 0.1 mM DTT, pH 8.0, and supplemented with an EDTA‐free protease inhibitor cocktail and lysozyme (0.2 μg mL^−1^). Cell lysis was performed by sonication, and the lysate was clarified by centrifugation at 25,000×*g* for 20 min at 4°C. DNA precipitation was achieved via the addition of streptomycin (1% final concentration) followed by centrifugation at 35,000×*g* for 30 min at 4°C. The resulting supernatant was filtered and loaded onto a Ni–NTA Sepharose column (GE Healthcare), pre‐equilibrated with 20 mM NaP, 150 mM NaCl, 0.1 mM DTT, 10 mM imidazole, pH 8.0. Proteins were eluted using a linear imidazole gradient from 10 to 500 mM. Fractions containing PaCysK or PaCysM were pooled, concentrated, and buffer‐exchanged into 20 mM NaP, 150 mM NaCl, 0.1 mM DTT, pH 8.5, using Vivaspin concentrators (Sartorius, Göttingen, DE). Protein purity was assessed by SDS‐PAGE and exceeded 95%. Each purification yielded approximately 150 mg of pure PaCysK and PaCysM per liter of bacterial culture. The extinction coefficients of monomeric PaCysK and PaCysM at 280 nm were calculated using the ProtParam tool (http://www.expasy.ch/tools/protparam.html) and were 18,512, and 24,472 M^−1^ cm^−1^, respectively. PLP content was determined by releasing the coenzyme in 0.1 M NaOH and measuring absorbance at 388 nm, using an extinction coefficient of  6,600 M^−1^ cm^−1^ (Gut et al., 2009).

The oligomeric state of the enzymes was assessed by size‐exclusion chromatography using a Superdex 200 Increase GL 10/300 column equilibrated in 50 mM Hepes, 150 mM NaCl, and 0.1 mM DTT, pH 8.5. Column calibration was performed according to the protocols in (Fernández‐Rodríguez et al., 2021; Fernández‐Rodríguez et al., 2023; Maresi et al., 2018).

### 4.3 Spectroscopic measurements

UV‐visible absorption spectra of each enzyme at a concentration of 15 μM were measured from 260 to 550 nm using a Jasco V‐750 UV–visible spectrophotometer. Measurements were carried out in 20 mM NaP, 150 mM NaCl and 0.1 mM DTT, pH 8.5 at 25°C.

Circular dichroism (CD) spectra were acquired on a Jasco J‐1500 CD spectropolarimeter equipped with a Peltier‐controlled thermostated cell holder (Conter et al., 2020). Briefly, Far‐UV CD spectra (190–260 nm) of PaCysK or PaCysM at 0.2 mg mL^‐1^ were measured using a quartz cuvette with a 0.1‐cm path length. Near‐UV‐visible spectra (250–600 nm) were recorded at 1 mg mL^−1^ protein concentration using a 1‐cm path length quartz cuvette at 25°C (Conter et al., 2023). For each scan, three accumulations were recorded, averaged, and baseline‐corrected using a buffer‐only blank. Thermal unfolding was monitored by measuring ellipticity at 222 nm over a temperature range of 15 to 100°C with a heating rate of 1.5°C min^−1^. Measurements were performed in a 0.1‐cm path length quartz cuvette with a protein concentration of 0.2 mg mL^−1^. All CD experiments were conducted in 20 mM NaP, pH 8.0.

### 4.4 Liquid chromatography mass spectrometry (LC–MS/MS)

Reactions involving either PaCysK or PaCysM for subsequent LC–MS/MS analysis were conducted in 10 mM Hepes, pH 8.0. Each reaction was carried out in a final volume of 50 μL at 37°C for 30 min, using 5 mM of each sulfur donor (either Na_2_S or Na_2_S_2_O_3_) in combination with 5–10 mM OAS, and 15 μM of the respective enzyme. After incubation, reactions were diluted to a final volume of 500 μL with deionized water, and the enzymes were removed by centrifugation using Amicon Ultra centrifugal filters with a 3 kDa molecular weight cut‐off (Sigma‐Aldrich). A 200 μL aliquot of the resulting filtrate, containing residual substrates and reaction products, was acidified with formic acid to a final concentration of 0.1% and subjected to LC–MS/MS analysis.

Analyses were performed as previously described (Pedretti et al., 2024), with minor modifications, using a TSQ Fortis Triple Quadrupole mass spectrometer (Thermo Scientific) coupled to an Ultimate 3000 HPLC system (Thermo Scientific). Product separation was achieved using a Luna C18(2) column (150 × 4.6 mm, 3 μm particle size, Phenomenex) under gradient elution. The mobile phase consisted of 0.1% formic acid in water (solvent A) and 0.1% formic acid in acetonitrile (solvent B). The chromatographic gradient was as follows: for positive mode, the flow rate was 0.4 mL min^−1^, with 2% B at 0 min, held for 2 min, then linearly increased to 20% B over 8 min, held at 90% B for 3 min, followed by a 5 min re‐equilibration period. For negative mode, the flow rate was also 0.4 mL min^−1^, with 20% B at 0 min, held for 1 min, then linearly increased to 60% B over 8 min, held at 90% B for 3 min, followed by a 5 min re‐equilibration period. Electrospray ionization (ESI) source parameters were as follows: spray voltage, +3500 V (for positive mode), −2500 V (for negative mode); ion transfer tube temperature, 300°C; vaporizer temperature, 350°C; sheath gas and auxiliary gas set to 50 and 10 (arbitrary units), respectively. Multiple reaction monitoring (MRM) was performed using nitrogen as the collision gas at a pressure of 1.5 mTorr. Two MRM transitions were selected for each analyte for reliable identification. Data acquisition and processing were carried out using Chromeleon software (version 7.2, Thermo Fisher).

### 4.5 Enzyme activity assays

TNB assay was performed as previously described (Tai et al., 1993). Briefly, the activities of PaCysK (15 μM) and PaCysM (1 μM) were measured at 25°C in 200 μL reaction mixtures containing 100 mM Hepes pH 8.0, 20 μM PLP, and OAS and TNB as substrates. OAS concentrations were varied from 0.1 to 20 mM, while TNB concentrations were varied from 0.01 to 0.1 mM.

The ninhydrin assay was performed as previously described (Gaitonde, 1967), with minor modifications. Briefly, PaCysK (final concentration: 25 nM–10 μM) or PaCysM (final concentration 30–50 nM) was added to a reaction mixture containing 20 mM NaP, pH 8.0, and either Na_2_S or Na_2_S_2_O_3_ (final concentration 0–30 mM) in combination with OAS (final concentration: 0–30 mM) as substrates. Each 250 μL reaction was incubated at 37°C for 1–3 min, depending on the enzyme and the specific reaction being tested. At pH 8.0, sulfide derived from Na_2_S is present predominantly as HS^−^, accounting for ~92.6% of total sulfide at 37°C, as estimated from the Henderson–Hasselbalch equation (pK_a1_ = 6.9; pK_a2_ = 12.7).

The reaction was quenched by the addition of 71.5 μL of 70% perchloric acid, followed by centrifugation at 19,000×*g* for 5 min at 5°C to remove the enzyme. A 250 μL aliquot of the resulting supernatant was collected and mixed with an equal volume of ninhydrin reagent, prepared by dissolving 250 mg of ninhydrin in 6 mL of 100% acetic acid and 4 mL of 0.6 M phosphoric acid. Samples were heated at 95°C for 5 min, cooled on ice for 5 min, and vortexed after dilution with 500 μL of 100% cold ethanol. Absorbance was measured at 560 nm. Negative controls, containing all assay components except the enzyme, were processed in parallel. Calibration curves were generated using either cysteine or S‐sulfocysteine standards, which were prepared and processed under the same conditions as the reaction samples.

Depending on whether the data displayed a sigmoidal or hyperbolic trend, kinetic parameters were determined by fitting to the Hill or Michaelis–Menten equation, respectively. In cases of substrate inhibition, data were fitted to a non‐hyperbolic model.

Temperature‐dependent loss of activity for PaCysK and PaCysM was assessed by measuring the residual enzymatic activity after incubating each protein at temperatures ranging from 37°C to 100°C for 10 min. For inhibition assays in the presence of the StP10 peptide, the enzyme was pre‐incubated at 37°C for 15 min with increasing concentrations of the peptide (0–1 mM), followed by a 1 min incubation on ice. The enzymatic reaction was then carried out as described above. Residual enzymatic activity was calculated from the initial reaction velocities and expressed as a percentage of the activity measured in the absence of peptide. These values were plotted against peptide concentration to determine the half‐maximal inhibitory concentration (IC_50_). The type of inhibition was determined from the pattern of the double‐reciprocal plots of 1/V_i_ against 1/[S] for the different peptide concentrations (0–25 μM). The *K*_*i*_ was determined by using the secondary plot of the slopes against the peptide concentrations which for competitive inhibition is linear, with the intersect on the x axis representing −*K*_*i*_.

### 4.6 Stopped flow experiments

Pre‐steady state kinetic measurements were carried out using a stopped‐flow apparatus (DX.17MV, Applied Photophysics, Leatherhead, UK) allowing rapid mixing of solutions in a 1:1 ratio. Experiments were carried out at 15°C in 20 mM NaP buffer, pH 8.0. In these measurements the light path was equal to 1.0 cm unless otherwise stated.

Formation of the α‐aminoacrylate intermediate (first half of the catalytic cycle, Figure 1b) was monitored by measuring the absorbance increase over time at 470 nm after mixing PaCysK or PaCysM (32 μM) with increasing OAS concentrations (from 0.250 mM up to 6 mM for PaCysK or 16 mM for PaCysM). For each condition, at least four individual kinetic traces were collected, averaged, and satisfactorily fitted to the following single exponential equation:

(1) $$A_{\left(t\right)}=A\cdot e^{\left(-k_{\mathrm{obs}}\cdot t\right)}+A_{\infty }$$

where *A*(*t*) represents the absorbance values at time *t*, *A* represents the amplitude of the collected trace, *k*_obs_ the observed rate constant, *t* the time of acquisition, and *A*_∞_ the absorbance values at infinite time.

The dependence of *k*_obs_ on the concentration of OAS was analyzed by fitting the data to a hyperbolic function:

(2) $$k_{\mathrm{obs}}=\frac{k_{\max}\;\left[\mathrm{OAS}\right]}{c_{50}+\left[\mathrm{OAS}\right]}$$

In this equation, *k*_max_ is the value of *k*_obs_ at saturating OAS and represents the rate constant for the β‐elimination of acetate from OAS to form the α‐aminoacrylate intermediate; *c*_50_ is the OAS concentration at which *k*_obs_ = 0.5 *k*_max_ (Tai et al., 2008).

The reaction of the α‐aminoacrylate intermediate with sulfur donors (second half of the catalytic cycle, Figure 1b) was kinetically investigated by multi‐wavelength stopped‐flow spectrophotometry, using the same stopped‐flow instrument above, equipped with a photodiode array detector enabling rapid spectral acquisition in the 200–700 nm range. Typically, PaCysK or PaCysM (29 μM) were pre‐incubated for at least 2 min with 20 or 40 mM OAS and then rapidly mixed with either Na_2_S (1 mM) or Na_2_S_2_O_3_ (10 mM for PaCysK, 6 mM for PaCysM). Spectral scans were collected from 3 to 20 ms for reactions with Na_2_S, and from 3 ms to 500 s (PaCysK) or to 100 ms (PaCysM) for reactions with Na_2_S_2_O_3_. All experiments were performed at 15°C in 20 mM NaP, pH 8.0. At pH 8.0 and 15°C, Na_2_S yields ~88.8% HS^−^.

### 4.7 Isothermal titration calorimetry (ITC)

Isothermal titration calorimetry (ITC) experiments were performed on a PEAQ‐ITC instrument (MicroCal, Northampton, MA; Conter et al., 2021; Pedretti et al., 2020). Stock solutions of StC10 or PaP9 peptide were prepared in deionized water and subsequently diluted in buffer containing 50 mM Hepes, 150 mM NaCl, 0.1 mM DTT, pH 8.5 to a final concentration of 500–800 μM. The protein solution, at a concentration of 60–80 μM in the same buffer, was titrated with 19 injections of the peptide (2 μL per injection). All experiments were performed at 25°C with a 150‐s interval between injections. Protein and ligand solutions were filtered and degassed prior to use. Reference titrations were conducted in the absence of the protein; the resulting heats of dilution were subtracted from the corresponding experimental datasets. Each titration was performed in triplicate, and results are reported as the mean ± standard error of the mean (SEM). Data analysis was conducted using MicroCal Origin software to determine the apparent dissociation constant (*K*_*d*_).

### 4.8 Bacterial strains and growth conditions

Bacterial strains used in this study are listed in Table S2. All *E. coli* and *P. aeruginosa* strains were routinely grown at 37°C in Lysogeny Broth (LB), or M9 Minimal Medium [0.77% (w/v) Na_2_HPO_4_•2H_2_O; 0.3% (w/v) KH_2_PO_4_; 0.05% (w/v) NaCl; 0.3% (w/v) NH_4_Cl; 0.025% (w/v) MgSO_4_•7H_2_O; 0.002% (w/v) CaCl_2_; 0.39% (w/v) glucose] in shaking conditions (200 rpm), or in LB supplemented with 1.5% (w/v) agar. When required, M9 medium recipe has been modified by substituting sulfate (1 mM MgSO_4_) with thiosulfate (30 mM Na_2_S_2_O_3_), and by adding an alternative source of magnesium (1 mM MgCl_2_). Antibiotics were added at the following concentrations: *E. coli*, 10 μg mL^−1^ tetracycline (Tc), or 30 μg mL^−1^ chloramphenicol (Cm); *P. aeruginosa*, 100 μg mL^−1^ Tc or 375 μg mL^−1^ Cm.

### 4.9 Recombinant DNA techniques

The plasmids and oligonucleotides used in this study are listed in Tables S3 and S4, respectively. Preparation of plasmid DNA, purification of DNA fragments, restriction enzyme digestion, ligation and transformation into *E. coli* DH5α or *E. coli* S17.1λ*pir* competent cells were performed by using standard procedures (Sambrock et al., 1989). Plasmid DNA was purified from bacterial cultures using the Wizard® Plus SC Minipreps DNA Purification System kit (Promega), according to the manufacturer's instructions. FastDigestTM restriction enzymes were purchased from Thermo Fisher Scientific. Ligation of DNA fragments obtained through digestion was performed using T4 DNA Ligase (Promega). DNA amplifications were performed by Polymerase Chain Reaction (PCR) using the GoTaq® Polymerase (Promega).

Plasmids were introduced into *P. aeruginosa* by transformation or by bi‐parental conjugation using *E. coli* S17.1λ*pir* as the donor strain (Sambrock et al., 1989). All plasmids generated in this study were checked by PCR and restriction analysis.

### 4.10 Construction of recombinant strains

Mutant strains of *P. aeruginosa* were generated *via* allelic exchange using plasmids derived from pDM4, following established protocols (Caruso et al., 2024; Milton et al., 1996). Details regarding the construction of these pDM4‐based plasmids are provided in Table S3. The plasmids were introduced into *P. aeruginosa* through conjugative transfer using *E. coli* S17.1λ*pir* as the donor strain (Sambrock et al., 1989). Selection of clones with chromosomal integration of the pDM4‐derived constructs was performed on LB agar plates containing 375 μg mL^−1^ chloramphenicol (Cm) and 15 μg mL^−1^ nalidixic acid (Nal). For the counter‐selection step, 10% (w/v) sucrose in LB was used. Confirmation of mutant strains was carried out by PCR.

### 4.11 Analysis of *P. aeruginosa* growth kinetics

Bacterial cultures were grown at 37°C with shaking (200 rpm) in M9 supplemented with 400 μM l‐cysteine. The medium was supplemented with 100 μg mL^−1^ Tc in the case of strains carrying pME6032 or its derivatives. After 16 h, cultures were washed twice in sterile saline [0.9% (w/v) NaCl] and diluted to an optical density at 600 nm (OD_600_) of 0.01 in M9 (in which 1 mM MgSO_4_ is the sole sulfur source), or in a modified M9 medium containing 30 mM Na_2_S_2_O_3_ and 1 mM MgCl_2_ in place of MgSO_4_. The M9 medium was supplemented with 0.5 mM IPTG for strains carrying the plasmid pME6032 or its derivatives. Two hundred μL aliquots of the resulting cultures were dispensed in 96‐well microtiter plates. OD_600_ values were recorded every hour for 20 h using an automated luminometer‐spectrophotometer plate reader Spark10M (Tecan).

### 4.12 Sequence and structural analysis

Sequence alignment was performed using Jalview (10.1093/bioinformatics/btp033), and the figure was prepared with ESPrit3.0 (https://doi.org/10.1093/nar/gku316).

AlphaFold models of PaCysK (AF‐Q9I0D3‐F1) and PaCysM (AF‐Q9I526‐F1) were obtained from the UniProt server. Structural superposition, analysis, and figure generation were carried out using UCSF ChimeraX (Meng et al., 2023).

## AUTHOR CONTRIBUTIONS

**Noemi Massa:** Investigation; writing – review and editing. **Flavia Catalano:** Investigation; writing – review and editing. **Silvia Fruncillo:** Investigation; writing – review and editing. **Francesca Troilo:** Investigation; writing – review and editing. **Marta Mellini:** Investigation; writing – review and editing. **Filippo Favretto:** Investigation; writing – review and editing. **Livia Leoni:** Writing – review and editing; funding acquisition. **Giordano Rampioni:** Conceptualization; funding acquisition; writing – review and editing; investigation. **Alessandro Giuffrè:** Conceptualization; funding acquisition; writing – review and editing; investigation. **Adele di Matteo:** Conceptualization; investigation; funding acquisition; writing – original draft; writing – review and editing; supervision. **Alessandra Astegno:** Conceptualization; funding acquisition; investigation; writing – review and editing; supervision; writing – original draft.

## Supporting information


**TABLE S1.** Steady‐state kinetic parameters of PaCysK and PaCysM at 25°C using OAS and TNB as substrates.
**TABLE S2.** Bacterial strains used in this study.
**TABLE S3.** Plasmids used in this study.
**TABLE S4.** Oligonucleotides used in this study.
**FIGURE S1.** Multiple sequence alignment of CysK and CysM.
**FIGURE S2.** Steady‐state enzyme kinetics of PaCysK and PaCysM using OAS and TNB as substrates.
**FIGURE S3.** Residual activity of PaCysK and PaCysM at increasing temperatures.
**FIGURE S4.** Near‐UV visible CD spectra of PaCysK and PaCysM.
**FIGURE S5.** Rapid‐scanning stopped‐flow spectra of the reaction between PaCysK or PaCysM and OAS.
**FIGURE S6.** AlphaFold models of PaCysM and PaCysK.
**FIGURE S7.** Electrostatic surface and structural comparison of the putative active sites of PaCysK and PaCysM with α‐aminoacrylate intermediate.
**FIGURE S8.** Multiple sequence alignment of CysE enzymes.

## Data Availability

The data that support the findings of this study are available from the corresponding author upon reasonable request.
