# Supplementary material for: Distinct contributions of O‐acetylserine sulfhydrylases to cysteine biosynthesis in Pseudomonas aeruginosa
Source: Protein Sci. 2026 Feb 12;35(3):e70498. doi: 10.1002/pro.70498 (PMC12895370; doi:10.1002/pro.70498)
Supplement: Supplementary file 1 — TABLE S1. Steady‐state kinetic parameters of PaCysK and PaCysM at 25°C using OAS and TNB as substrates. TABLE S2. Bacterial strains used in this study. TABLE S3. Plasmids used in this study. TABLE S4. Oligonucleotides used in this study. FIGURE S1. Multiple sequence alignment of CysK and CysM. FIGURE S2. Steady‐state enzyme kinetics of PaCysK and PaCysM using OAS and TNB as substrates. FIGURE S3. Residual activity of PaCysK and PaCysM at increasing temperatures. FIGURE S4. Near‐UV visible CD spectra of PaCysK and PaCysM. FIGURE S5. Rapid‐scanning stopped‐flow spectra of the reaction between PaCysK or PaCysM and OAS. FIGURE S6. AlphaFold models of PaCysM and PaCysK. FIGURE S7. Electrostatic surface and structural comparison of the putative active sites of PaCysK and PaCysM with α‐aminoacrylate intermediate. FIGURE S8. Multiple sequence alignment of CysE enzymes. [file PRO-35-e70498-s001.pdf]

Supplementary Material

Distinct contributions of O-acetylserine sulfhydrylases to cysteine biosynthesis in *Pseudomonas aeruginosa*

Noemi Massa [a, #], Flavia Catalano [b, #], Silvia Fruncillo [a], Francesca Troilo [b], Marta Mellini [c], Filippo Favretto [a], Livia Leoni [c], Giordano Rampioni [c,d], Alessandro Giuffrè [b], Adele di Matteo [b, *], Alessandra Astegno [a, *]

[a] *Department of Biotechnology, University of Verona, Verona, Italy.*
[b] *CNR Institute of Molecular Biology and Pathology, Rome, Italy.*
[c] *Department of Science, University Roma Tre, Rome, Italy*
[d] *IRCCS Fondazione Santa Lucia, Rome, Italy*

[#] These authors contributed equally.

*Corresponding authors. Alessandra Astegno, e-mail: alessandra.astegno@univr.it; Adele di Matteo, e-mail: adele.dimatteo@cnr.it

LIST OF SUPPLEMENTARY CONTENT

SUPPLEMENTARY METHODS
Rapid-scanning stopped-flow experiments.

SUPPLEMENTARY TABLES
Table S1. Steady-state kinetic parameters of PaCysK and PaCysM at 25 °C using OAS and TNB as substrates.
Table S2. Bacterial strains used in this study.
Table S3. Plasmids used in this study.
Table S4. Oligonucleotides used in this study.

SUPPLEMENTARY FIGURES
Figure S1. Multiple sequence alignment of CysK and CysM.
Figure S2. Steady-state enzyme kinetics of PaCysK and PaCysM using OAS and TNB as substrates.
Figure S3. Residual activity of PaCysK and PaCysM at increasing temperatures.
Figure S4. Near-UV visible CD spectra of PaCysK and PaCysM.
Figure S5. Rapid-scanning stopped-flow spectra of the reaction between PaCysK or PaCysM and OAS.
Figure S6. AlphaFold models of PaCysM and PaCysK.
Figure S7. Electrostatic surface and structural comparison of the putative active sites of PaCysK and PaCysM with α-aminoacrylate intermediate.
Figure S8. Multiple sequence alignment of CysE enzymes.

SUPPLEMENTARY METHODS

Rapid-scanning stopped-flow experiments

Rapid-scanning stopped-flow experiments were carried out using a DX.17MV spectrophotometer (Applied Photophysics, Leatherhead, UK) equipped with a diode-array detector. The enzymes (32 μM) were rapidly mixed with 1 mM OAS. Spectra were collected in the 300 - 600 nm range at the following time point after mixing 12, 24, 26, 48, 60, 192, 204, 216, 228, 240 ms. Spectra at time zero (t_0) correspond to the enzyme in the absence of substrate. All experiments were conducted in 20 mM sodium phosphate buffer, pH 8.0, at 15 °C. Differential spectra ($\Delta A = A_{tx} - A_{t_0}$) were calculated, where A_{t_0} represents the spectrum prior to substrate addition and A_{tx} spectra recorded at subsequent reaction times.

SUPPLEMENTARY TABLES

Table S1. Steady-state kinetic parameters of PaCysK and PaCysM at 25 °C using OAS and TNB as substrates. Values represent mean ± SEM from ≥ 3 independent measurements, obtained using at least two different protein batches.

	**PaCysK**	**PaCysM**	**StCysK***	**StCysM***
$\mathbf{k_{cat}}$ (s^{-1})	0.02 ± 0.01	1.5 ± 0.2	0.56 ± 0.08	8 ± 2
$\mathbf{K_m}$ **(OAS)** (mM)	0.50 ± 0.03	0.09 ± 0.03	15 ± 3	0.19 ± 0.08
$\mathbf{K_m}$ **(TNB)** (mM)	0.04 ± 0.02	0.06 ± 0.02	0.6 ± 0.1	0.43 ± 0.09
$\boldsymbol{k_{cat}/K_m}$ **(OAS)** (M^{-1} s^{-1})	40 ± 20	(1.7 ± 0.6) × 10^4	37 ± 5	(4.1 ± 1.9) ×10^4
$\boldsymbol{k_{cat}/K_m}$ **(TNB)** (M^{-1} s^{-1})	500 ± 350	(2.5 ± 0.9) ×10^4	950 ± 55	(1.8 ± 0.1) ×10^4

*For comparison, kinetic parameters of *Salmonella enterica* serovar Typhimurium homologs (StCysK and StCysM) are reported [1].

Table S2. Bacterial strains used in this study

**Strains**	**Relevant characteristics**	**Reference/Source**
E. coli		
DH5α	Cloning strain	[S2]
S17.1λ*pir*	Conjugative strain for suicide plasmids	[S3]
P. aeruginosa		
PAO1	Reference isolate, wild type, ATCC 15692 type strain	American Type Culture Collection
Δ*cysK*	PAO1 derivative strain carrying a deletion of the *cysK* gene (PA2709), obtained by allelic exchange using the plasmid pDM4Δ*cysK* (**Table S2**).	This study
Δ*cysM*	PAO1 derivative strain carrying a deletion of the *cysM* gene (PA0932), obtained by allelic exchange using the plasmid pDM4Δ*cysM* (**Table S2**).	This study
Δ*cysKM*	Δ*cysM* derivative strain carrying a deletion of the *cysK* gene (PA2709), obtained by allelic exchange using the plasmid pDM4Δ*cysK* (**Table S2**).	This study

Table S3. Plasmids used in this study.

Plasmids	Relevant characteristics and plasmids construction	Reference/Source
pDM4	Suicide vector; *sacBR*; *oriR6K*; Cm^R.	[S4]
pME6032	IPTG inducible expression vector, *lacI*^{q}-P_{tac}, Tc^R.	[S5]
pDM4Δ*cysK*	pDM4-derived plasmid for the generation of the Δ*cysK* mutant strain; Cm^R. It contains the DNA fragments encompassing the upstream region of the *cysK* gene originated with primers FW*cysK*UP and RV*cysK*UP (**Table S3**), and the downstream region of the *cysK* gene originated with primers FW*cysK*DW and RV*cysK*DW (**Table S3**), cloned in pDM4 by XhoI-XbaI restriction.	This study
pDM4Δ*cysM*	pDM4-derived plasmid for the generation of the Δ*cysM* mutant strain; Cm^R. It contains the DNA fragments encompassing the upstream region of the *cysM* gene originated with primers FW*cysM*UP and RV*cysM*UP (**Table S3**), and the downstream region of the *cysM* gene originated with primers FW*cysM*DW and RV*cysM*DW (**Table S3**), cloned in pDM4 by XhoI-XbaI restriction.	This study
pME-*cysK*	pME6032 derivative carrying the coding sequence of *cysK* downstream of the IPTG-inducible P_{tac} promoter. The *cysK* gene was amplified from PAO1 genome with the primer pair *cysK*-FW and *cysK*-RV (**Table S3**), and cloned in pME6032 by EcoRI-KpnI restriction.	This study
pME-*cysM*	pME6032 derivative carrying the coding sequence of *cysM* downstream of the IPTG-inducible P_{tac} promoter. The *cysM* gene was amplified from PAO1 genome with the primer pair *cysM*-FW and *cysM*-RV (**Table S3**), and cloned in pME6032 by EcoRI-KpnI restriction.	This study

Table S4. Oligonucleotides used in this study.

Name	Sequence (5'-3')[a]	Restriction site
FW*cysK*UP	CCGCTCGAGGAGCTGCGGACGCTCGAA	XhoI
RV*cysK*UP	CCGGAATTCCTTGGCCAGGATGGTGACG	EcoRI
FW*cysk*DW	CCGGAATTCACGGCCTGTTCAGCGAACA	EcoRI
RV*cysK*DW	GCTCTAGAGAGCTTCTCGGCGAAGCT	XbaI
FW*cysM*UP	CCGCTCGAGGGCCGCCAGGAGCCGC	XhoI
RV*cysM*UP	CCCAAGCTTAGGGGTATTGCCAACGCAG	HindIII
FW*cysM*UDW	CCCAAGCTTCCTGTCTTCCGGCGTCTAT	HindIII
RV*cysM*DW	GCTCTAGAACGGAAGCCGACATCCAGA	XbaI
cysK-FW	CCGGAATTCATGAGCCGCATCTTCGC	EcoRI
cysK-RV	CGGGGTACCTTACTGGGTCAGTTCCTGTT	KpnI
cysM-FW	CCGGAATTCATGACCGTGCAGTACCC	EcoRI
cysM-RV	CGGGGTACCTCAGCGCGGGTCATAGA	KpnI

[a] Restriction sites are underlined in the primer sequences.

SUPPLEMENTARY FIGURES

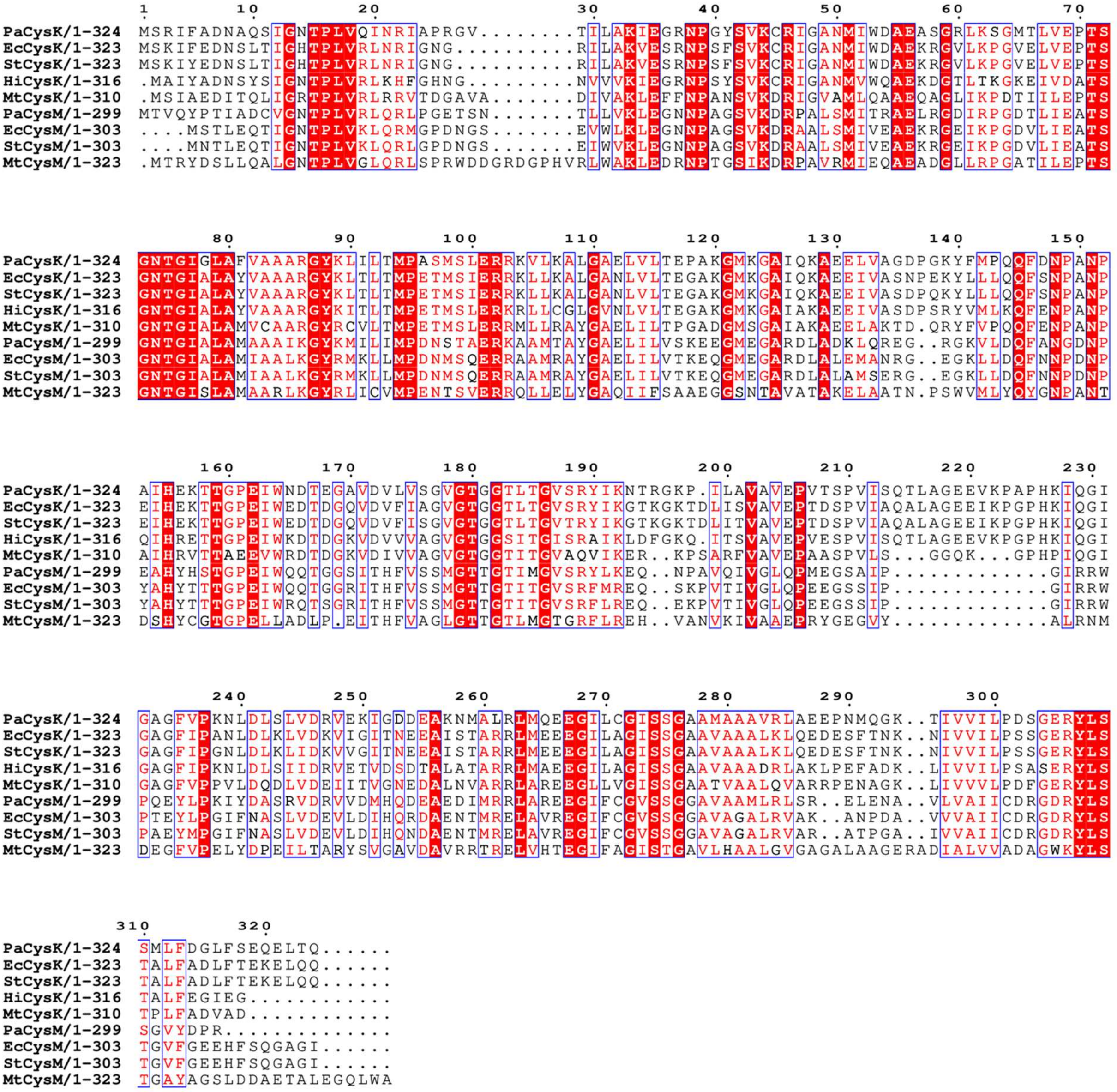

Figure S1. Multiple sequence alignment of CysK and CysM. *P. aeruginosa* CysK (PaCysK, UniProt ID: Q9I0D3), *E. coli* CysK (EcCysK, UniProt ID: P0ABK5), *Salmonella enterica* serovar Typhimurium CysK (StCysK, UniProt ID: P0A1E3), *H. influenzae* CysK (HiCysK, UniProt ID: P45040), *M. tuberculosis* CysK1 (MtCysK1, UniProt ID: P9WP55), *P. aeruginosa* CysM (PaCysM, UniProt ID: Q9I526), *E. coli* CysM (EcCysM, Uniprot ID: P16703), *S. enterica* ser. Typhimurium CysM (StCysM, UniProt ID: P29848), *M. tuberculosis* CysM (MtCysM, UniProt ID: P9WP53).

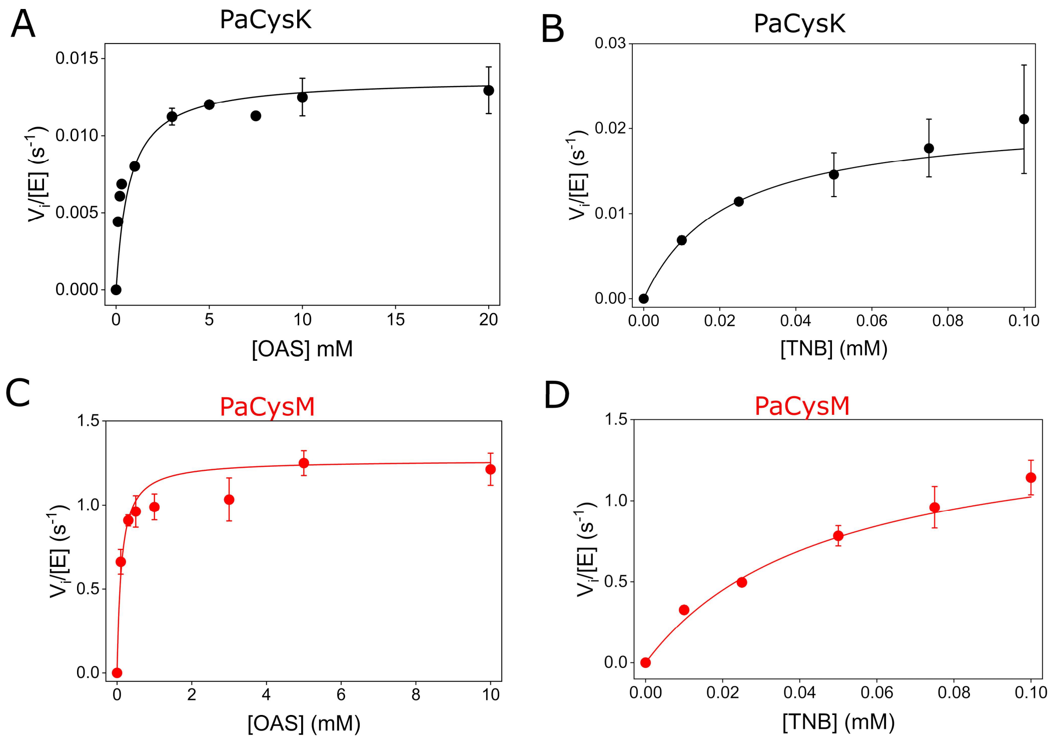

Figure S2. Steady-state enzyme kinetics of PaCysK and PaCysM using OAS and TNB as substrates. (A-B) PaCysK reaction rates as a function of OAS concentration (**A**) and TNB concentration (**B**). (**C-D**) PaCysM reaction rates as a function of OAS concentration (**C**) and TNB concentration (**D**). Kinetic parameters derived from these experiments are summarized in **Table S1**.

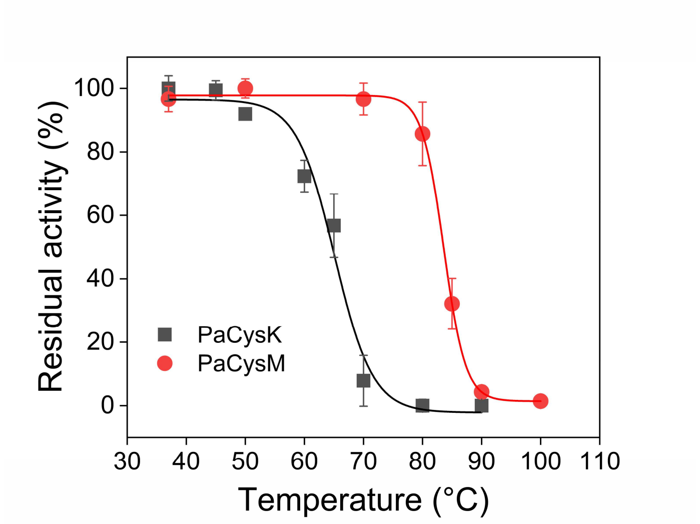

Figure S3. Residual activity of PaCysK and PaCysM at increasing temperatures. Activity was measured using 10 mM OAS with 0.5 mM Na_2S for PaCysK, and 5 mM Na_2S for PaCysM, after incubating the enzymes for 10 minutes at various temperatures.

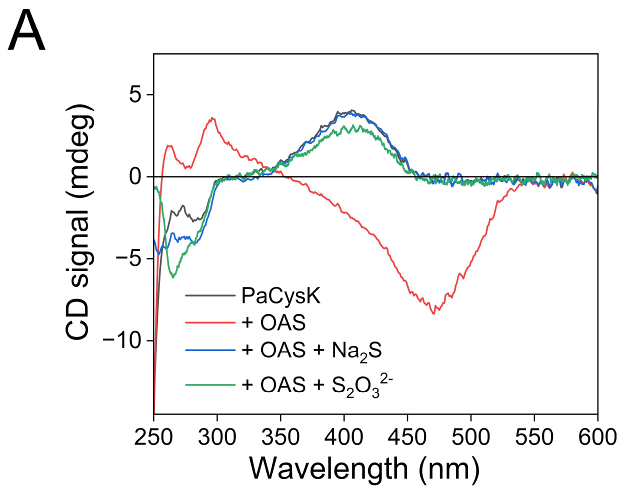

Figure S4. Near-UV visible CD spectra of PaCysK and PaCysM. CD Spectra of 1 mg mL^{-1} PaCysK (A) and PaCysM (B) alone (black), after addition of OAS (red), and after subsequent addition of Na_2S (blue) or $Na_2S_2O_3$ (green) to the protein-OAS complex.

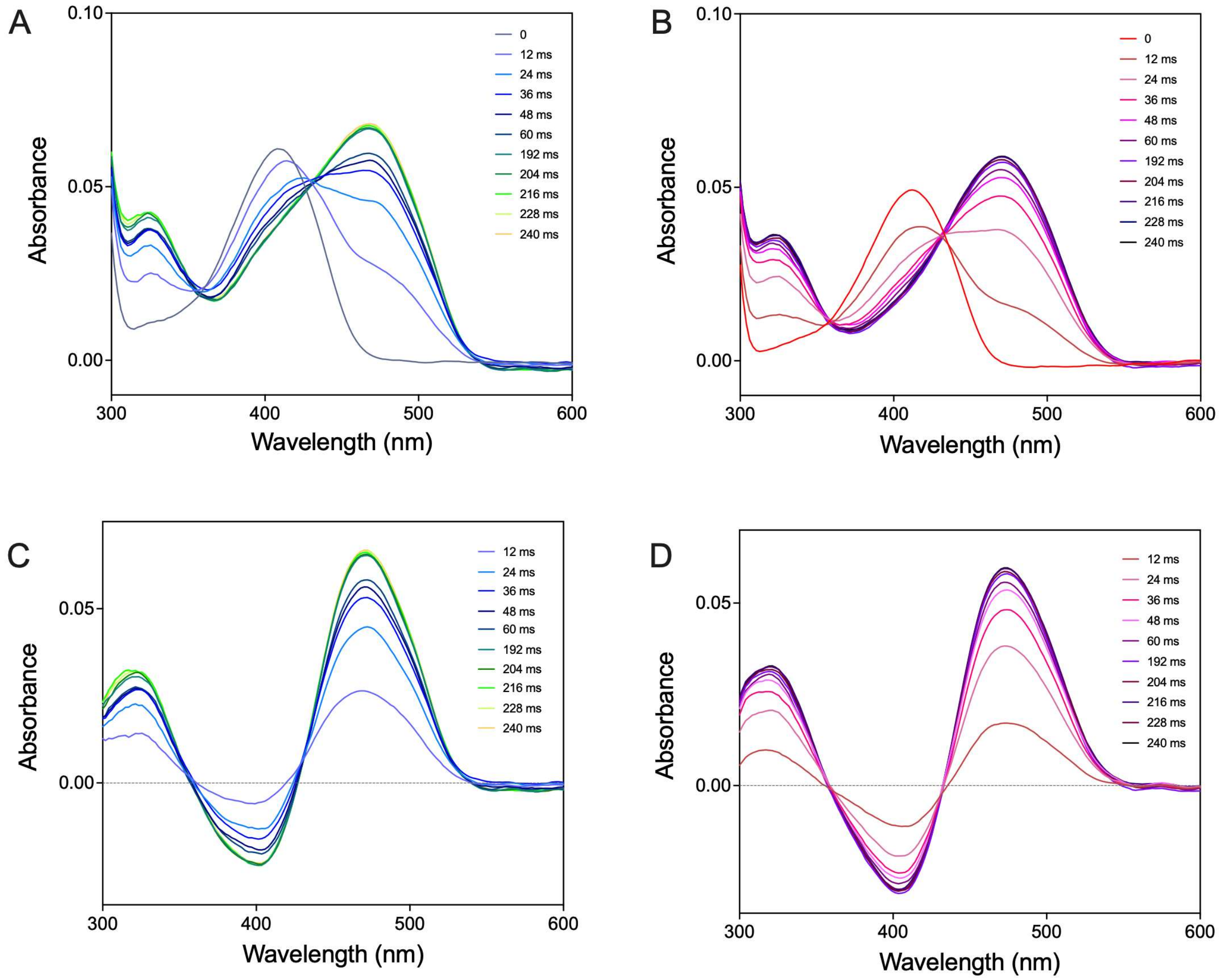

Figure S5. (**A, B**) Time-resolved absorption spectra acquired after stopped-flow mixing 32 µM PaCysK (**A**) or PaCysM **(B)** with 1 mM OAS. Spectrum t_0 corresponds to the enzyme before mixing, while subsequent spectra were recorded at the indicated times after mixing with OAS. (**C, D**) Corresponding difference spectra ($\Delta A = At_x - At_0$, where At_0 is the spectrum of the enzyme before mixing and At_x refers to the spectra collected at the indicated reaction times) for PaCysK (**C**) and PaCysM (**D**). The spectra highlight the absence of a single isosbestic point in the earliest time windows, indicating the transient formation of a short-lived intermediate during the first milliseconds of the reaction. In the spectra collected at 12 and 24 ms after mixing, the signals between 370 and 500 nm reflect a combination of the external Schiff base and the α-aminoacrylate species.

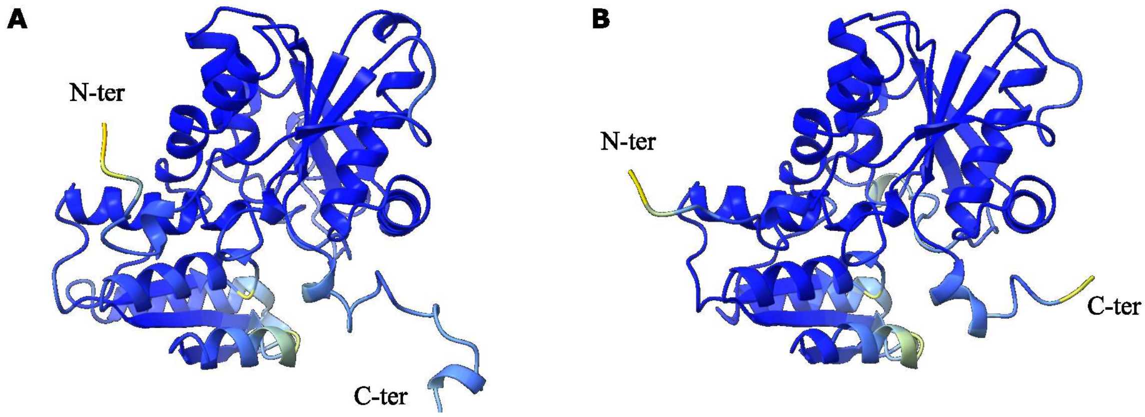

Figure S6. AlphaFold models of PaCysM and PaCysK. AlphaFold models of PaCysM (AF-Q9I0D3-F1) (A) and PaCysK (AF-Q9I526-F1) (B), colored according to the predicted Local Distance Difference Test (pLDDT) score.

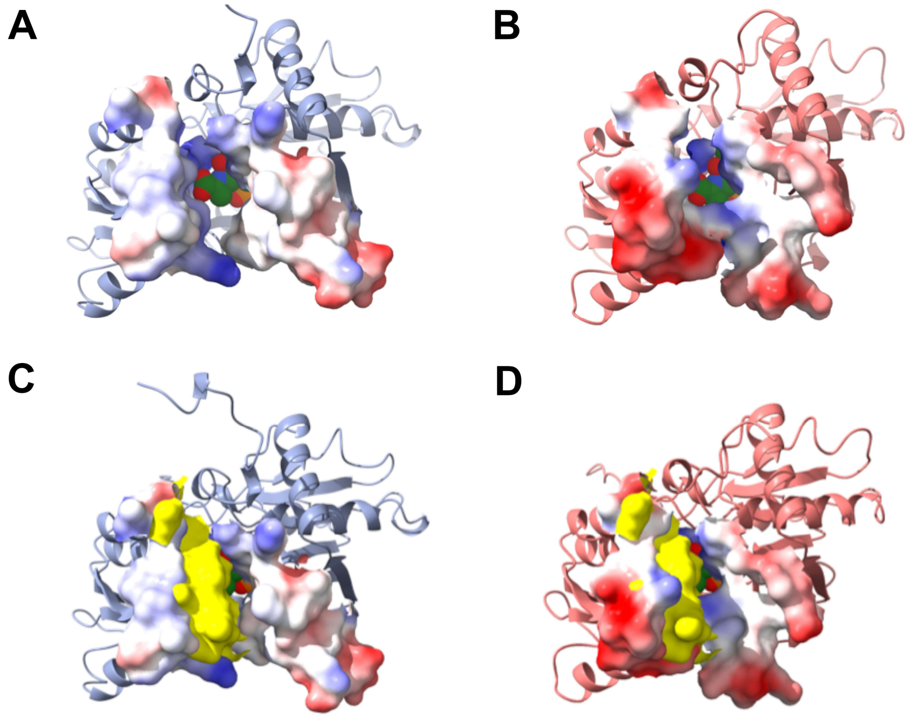

Figure S7. Electrostatic surface and structural comparison of the putative active sites of PaCysK and PaCysM with α-aminoacrylate intermediate. Electrostatic surface representation of the putative active site region of **(A)** AF-Q9I0D3-F1 (PaCysK, grey) and **(B)** AF-Q9I526-F1 (PaCysM, pink). The α-aminoacrylate intermediate, positioned based on structural alignment with the reference model of MtCysK (PDB: 2Q3D), is shown as spheres. On the right, the segment of MtCysK1 that folds over the active site upon interaction with the α-aminoacrylate has been superimposed onto the PaCysK **(C)** and PaCysM **(D)** models, suggesting a possible mode of active site closure during the formation of the complex with α-aminoacrylate in *P. aeruginosa*.

```
PaCysE1        ......................................................................
PaCysE2     1  ...................................................MRAALMECCSPQLVERIVA
StCysE      1  MPCEELEIVWKNIKAEARALADCEPMLASFYHATLLKHENLGSALSYMLANKLASPIMPAIAIREVVEEA
EcCysE      1  MSCEELEIVWNNIKAEARTLADCEPMLASFYHATLLKHENLGSALSYMLANKLSSPIMPAIAIREVVEEA
HiCysE      1  MTLD....VWQHIRQEAKELAENEPMLASFFHSTILKHQNLGGALSYLLANKLANPIMPAISLREIIEEA
MtCysE         ......................................................................

PaCysE1     1  ......MFERVREDIQSVFHRDPAAR.NALEVLTCYPGLHAVWLHRLAHGLWTSGW.........KWLAR
PaCysE2    20  HSMFEEARRWCEEDLQAFASKDPAAQGRTSDIAFGYSSFKAVLHYRLSHMLCLRSTSEGDPERALEATAL
StCysE     71  YAADPEMIASAACDIQAVRTRDPAVD.KYSTPLLYLKGFHALQAYRIGHWLWNKGR.........RALAI
EcCysE     71  YAADPEMIASAACDIQAVRTRDPAVD.KYSTPLLYLKGFHALQAYRIGHWLWNQGR.........RALAI
HiCysE     67  YQSNPSIIDCAACDIQAVRHRDPAVE.LWSTPLLYLKGFHAIQSYRITHYLWNQNR.........KSLAL
MtCysE      1  ......MLTAMRGDIRAARERDPAAP.TALEVIFCYPGVHAVWGHRLAHWLWQRGA.........RLLAR

PaCysE1    55  LVSNFGRWMTGIEIHPGARIGRRFFIDHGMGIVIGETAEIGDDVTLYQGVTLGGTTWNK...GKRHPTLG
PaCysE2    90  LVSSRGKLLSGAEIHPRCKIGSRFILDHGHGTVIGETAVIGDDCYILGGVVLGATGISANPAGKRHPTIG
StCysE    131  FLQNQVSVSFQVDIHPAAKIGRGIMLDHATGIVVGETAVIEDDVSILQSVTLGGTGKTS...GDRHPKIR
EcCysE    131  FLQNQVSVTFQVDIHPAAKIGRGIMLDHATGIVVGETAVIENDVSILQSVTLGGTGKSG...GDRHPKIR
HiCysE    127  YLQNQISVAFDVDIHPAAKIGHGIMFDHATGIVVGETSVIENDVSILQGVTLGGTGKES...GDRHPKVR
MtCysE     55  AAAEFTRILTGVDIHPGAVIGARVFIDHATGVVIGETAEVGDDVTIYHGVTLGGSGMVG...GKRHPTVG

PaCysE1   122  NNVVVGAGAKVLGPFTVGEGAKVGSNAVVTKEVPPGATVVGIPGRIIMREDSEQQAKRQAMAEKLGFDAY
PaCysE2   160  SRVQIGAFTRVLGDIAIGDDVFVGPHCVIKDDIPVGSVVTLRSELQVIR.....................
StCysE    198  EGVMIGAGAKILGNIEVGRGAKIGAGSVVLQPVPPHTTAAGVPARIVGKPGSD.................
EcCysE    198  EGVMIGAGAKILGNIEVGRGAKIGAGSVVLQPVPPHTTAAGVPARIVGKPDSD.................
HiCysE    194  EGVMIGAGAKILGNIEVGKYAKIGANSVVLNPVPEYATAAGVPARIVSQDKAA.................
MtCysE    122  DRVIIGAGAKVLGPIKIGEDSRIGANAVVVKPVPPSAVVVGVPGQVIGQSQPSPGGP.........FDWR

PaCysE1   192  GVSQDMPDPVARAIGQLLDHLQAVDGRLEGMCQALTALGSDYCAKDLPVLREEDFAGVKDEDGNPAA
PaCysE2   209  ..............GPHIVQQLQPAATQTQIQPMEAS..............................
StCysE    251  ..............KPSMDMDQHFNGIHHTFEYGDGI..............................
EcCysE    251  ..............KPSMDMDQHFNGINHTFEYGDGI..............................
HiCysE    247  ..............KPAFDMNQYFIGIDDGMNLN..I..............................
MtCysE    183  .....LPD....LVGASLDSLLTRVARLEALGGGPQAAG.........VIRPPE.AGIWHGEDFSI.
```

Figure S8. Multiple sequence alignment of CysE from different bacteria. *P. aeruginosa* CysE1 (PaCysE1, Uniprot ID: Q9HXI6), *P. aeruginosa* CysE2 (PaCysE2, Uniprot ID: Q9I210). *Salmonella enterica* serovar Typhimurium CysE (StCysE, Uniprot ID: P29847), *E. coli* CysE (EcCysE, Uniprot ID: P0A9D4), *H. influenzae* CysE (HiCysE, Uniprot ID: P43886), *M. tuberculosis* CysE (MtCysE, Uniprot ID: P95231).

References

S1. Tai, C.H., S.R. Nalabolu, T.M. Jacobson, D.E. Minter, and P.F. Cook, *Kinetic mechanisms of the A and B isozymes of O-acetylserine sulfhydrylase from Salmonella typhimurium LT-2 using the natural and alternate reactants.* Biochemistry, 1993. **32**(25): p. 6433-6442.

S2. Grant, S.G., J. Jessee, F.R. Bloom, and D. Hanahan, *Differential plasmid rescue from transgenic mouse DNAs into Escherichia coli methylation-restriction mutants.* Proc Natl Acad Sci U S A, 1990. **87**(12): p. 4645-9.

S3. Simon, R., U. Priefer, and A. Pühler, *A Broad Host Range Mobilization System for In Vivo Genetic Engineering: Transposon Mutagenesis in Gram Negative Bacteria.* Bio/Technology, 1983. **1**(9): p. 784-791.

S4. Milton, D.L., R. O'Toole, P. Horstedt, and H. Wolf-Watz, *Flagellin A is essential for the virulence of Vibrio anguillarum.* J Bacteriol, 1996. **178**(5): p. 1310-9.

S5. Heeb, S., C. Blumer, and D. Haas, *Regulatory RNA as mediator in GacA/RsmA-dependent global control of exoproduct formation in Pseudomonas fluorescens CHA0.* J Bacteriol, 2002. **184**(4): p. 1046-56.
